# Equity and inclusion in UK adult social care: a systematic review of trials

**DOI:** 10.1186/s12913-026-14347-y

**Published:** 2026-03-17

**Authors:** Flora Kaminski, Lucy Smith, Hajira Dambha-Miller

**Affiliations:** University of Southampton, Southampton, UK

**Keywords:** Social care, Interventions, Equity, Core20Plus5, Progress plus

## Abstract

**Background:**

Equity is a key priority in UK health and social care policy, yet its integration into adult social care trials remains unclear. Without adequate inclusion or analysis of underserved populations, interventions risk reinforcing health inequalities.

**Aim:**

To examine how equity-relevant characteristics - defined by CORE20PLUS5 and PROGRESS-Plus -are reported, categorised, and analysed in UK trials of adult social care interventions.

**Methods:**

A systematic review of five databases (PubMed, MEDLINE, Embase, CINAHL, Web of Science) was conducted for studies published between January 2015 and June 2025. Eligible studies involved UK-based adult populations (≥ 18 years) and used trial or quasi-experimental designs. Data were narratively synthesised due to heterogeneity. Study quality was assessed using Joanna Briggs Institute tools.

**Results:**

Fourteen studies met inclusion criteria. All reported at least one equity characteristic, but reporting was inconsistent. Ethnicity was often grouped into broad categories, limiting subgroup analysis. Few studies targeted underserved populations or designed interventions to address inequity. Reporting gaps included gender identity, sexual orientation, immigration status, and literacy.

**Conclusions:**

Equity considerations are limited in UK adult social care trials. Future research should embed equity frameworks, improve reporting detail, and conduct subgroup analyses to ensure interventions are inclusive and effective across all population groups.

**Supplementary Information:**

The online version contains supplementary material available at 10.1186/s12913-026-14347-y.

## Introduction

The UK health and social care system is experiencing mounting pressures. An ageing population with complex needs and increasing disability among working-age adults are driving higher demand for services [1, 2]. Chronic workforce shortages and sustained underfunding exacerbate these challenges, leaving many individuals with unmet or “hidden” care needs [3]. In 2023, nearly 2 million requests for social care support were made by working-age adults, yet only half were granted [4], while around 2 million older adults reported not receiving the care they required [5]. Hidden need is thought to be substantial: under the Care Act 2014, many eligible individuals are not receiving formal support [6]. For example, the Social Mobility Commission estimates that 1.5 million disabled adults in England remain without care despite being eligible [7]. These inequities arise from multiple, interacting determinants-unequal access to services, variation in quality and experience of care, differences in health behaviours, and wider social and economic factors such as housing, education, employment, and income. Collectively, these drivers contribute to premature mortality, reduced quality of life, and increasing strain on the system [8–10].

**Fig. 1:**
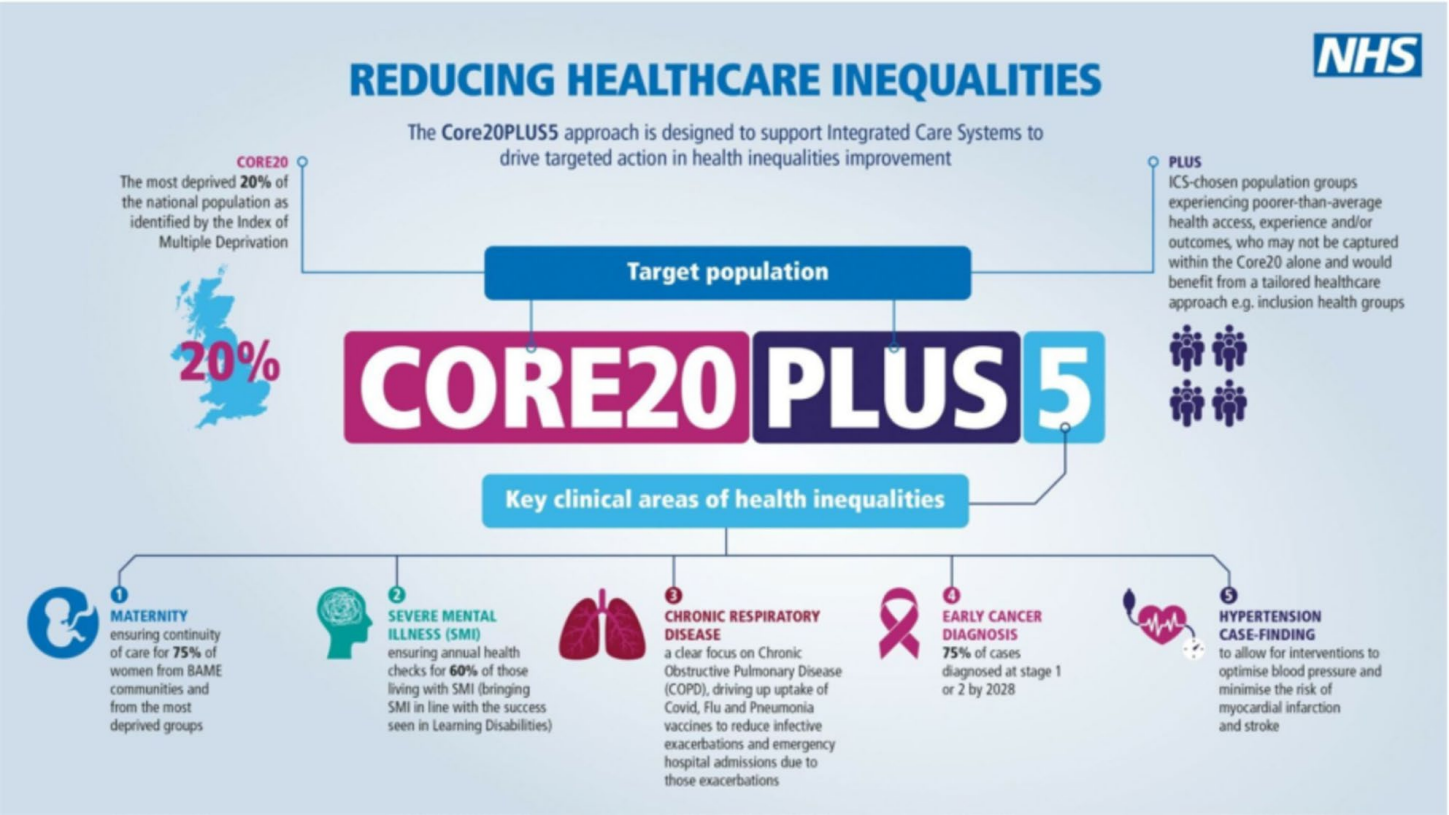
NHS England’s CORE20PLUS5 framework for reducing healthcare inequalities (image from source) [13]

Although equity is increasingly recognised in social care policy, the extent to which it is embedded in the evaluation of adult social care interventions is unclear. The NIHR INCLUDE guidance highlights the need for research that reflects the needs of underserved populations [11, 12]. NHS England’s CORE20PLUS5 framework (Fig. 1) provides a structured approach to addressing inequities by targeting three components: the most deprived 20% of the national population as defined by the Index of Multiple Deprivation (CORE20); additional groups who experience poorer-than-average access, experience, or outcomes of care (PLUS); and five clinical priority areas linked to the largest gaps in health outcomes (PLUS5) [13]. This framework has been widely adopted in policy and practice as a practical tool for embedding equity considerations into service design and evaluation.

**Fig. 2:**
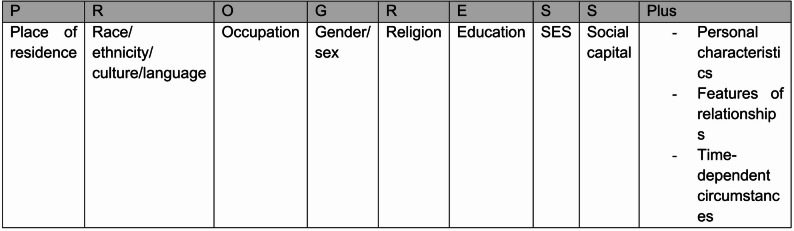
Description of progress-plus acronym. (adapted from 14)

**Fig. 3:**
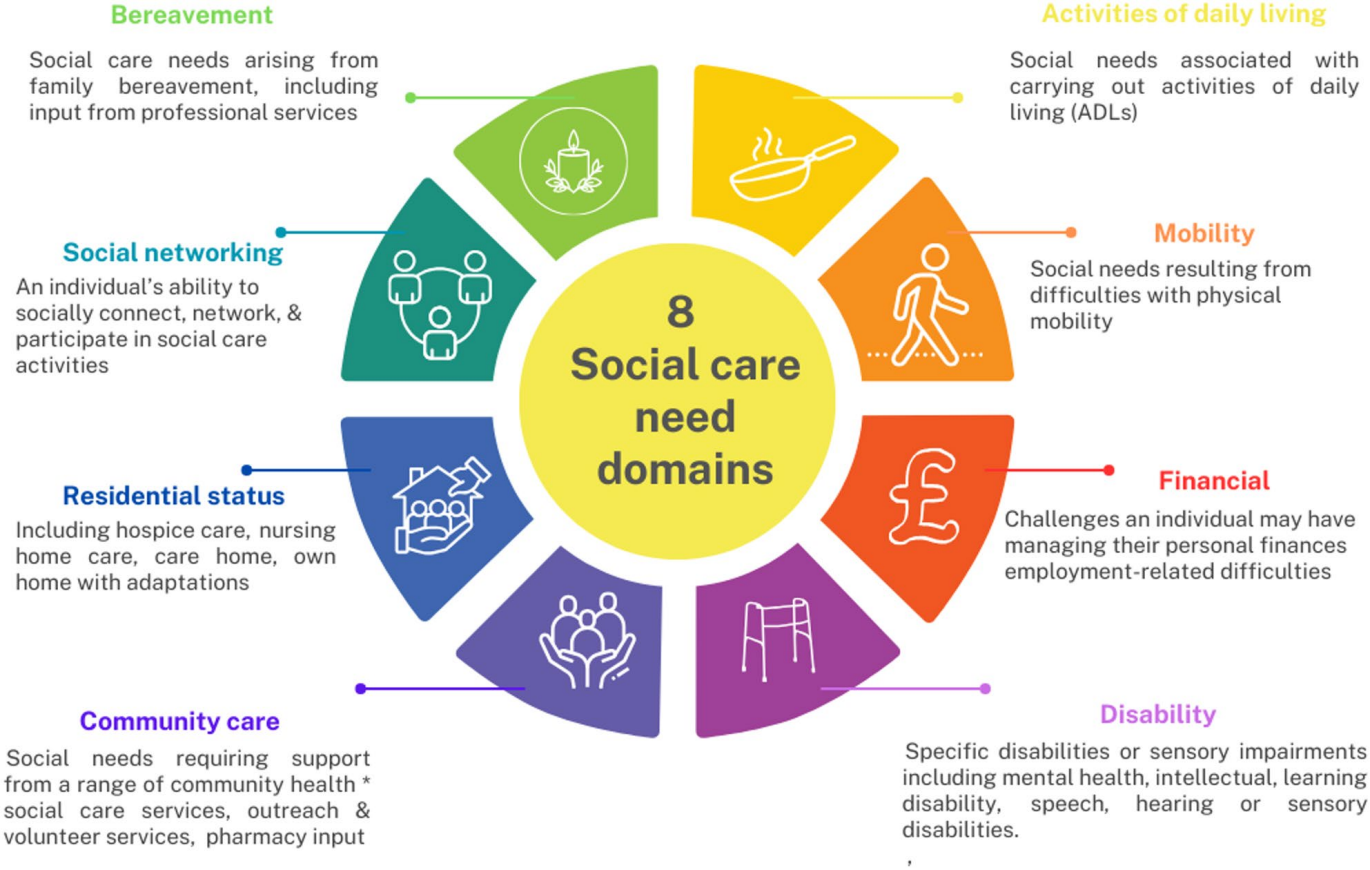
The eight social need domains derived from the framework developed by Issac et al. [14]

The PROGRESS-Plus framework (Fig. 2), endorsed by the Campbell and Cochrane Equity Methods Group, complements this by specifying social stratifiers that should be considered in research and evidence synthesis. PROGRESS represents Place of residence, Race/ethnicity, Occupation, Gender/sex, Religion, Education, Socioeconomic status, and Social capital, with “Plus” referring to additional context-specific characteristics such as age, disability, or sexual orientation [14]. This framework has been integrated into systematic review methodology through tools such as PRISMA-Equity and the Cochrane Handbook and provides a standardised lens for assessing equity impacts. More recently, Isaac et al. (2025) proposed a framework of eight domains of social care need (Fig. 3), offering a multidimensional structure for operationalising and comparing needs across populations [Appendix A Supplementary Table 1] [15]. While equity-focused systematic reviews in health research have demonstrated the value of these approaches [16, 17], no review has explicitly applied an equity lens to adult social care interventions. This review therefore aims to synthesise how equity-relevant characteristics, as defined by the CORE20PLUS5 and PROGRESS-Plus frameworks, are reported, categorised, and analysed in UK trials of adult social care interventions across the eight social care need domains described by Isaac et al. (2025), and to consider the implications for advancing equity in health and social care research.

## Method

### Design

A systematic review was reported using the Preferred Reporting Items for Systematic Reviews and Meta-Analyses (PRISMA) 2020 checklist to ensure a transparent and rigorous approach (see Appendix B for the completed checklist Supplementary Table 2) [18]. The protocol was prospectively registered with the International Prospective Register of Systematic Reviews (PROSPERO) on July 1st 2025 (ID: 1089898).

## Study selection criteria and justification

Inclusion and exclusion criteria for studies were defined prior to database searching. Detailed criteria are discussed below and summarised in Table 1.

**Table 1:** Inclusion and exclusion criteria applied during study selection

	Inclusion criteria	Exclusion criteria
Population	• Adults aged 18 years and over living in the UK	• Children under 18 years• Adults that do not live in the UK
Intervention	• Adult social care interventions targeting one or more of the eight domains of social care(15)	Interventions targeting needs not directly related to social care
Comparators	• All comparators (including usual care, no intervention or alternative social care intervention)	• No exclusion criteria
Study design	• Randomised controlled trials, non-randomised controlled trials with a comparator group • Mixed-method studies where one element is a trial	• Observational studies • Qualitive studies • Descriptive studies • Systematic reviews and meta-analyses • Non-experimental designs (protocols, conference abstracts)
Publication type	• Peer-reviewed journals	• Grey literature including conference abstracts and protocols
Publication characteristics	• English language • 2015 to present	• Language other than English

### Search strategy

#### Databases

Five electronic databases were systematically searched: PubMed, MEDLINE (Ovid), Embase, CINAHL, and Web of Science, and grey literature was searched from January 2015 up to 14 June 2025.

#### Search terms

The search strategy was developed using guidance from the Cochrane Handbook for Systematic Reviews and was refined further with an information specialist (For example:

“adult/ or (adult* or “working age” or older or elderly).ti,* ab*,* kw*,* kf.*

*United Kingdom/ or (UK or “United Kingdom” or Britain or Great Britain or England or English or Scotland or Scottish or Wales or Welsh or “Northern Ireland” or “Northern Irish”).ti*,* ab*,* kw*,* kf.*

(intervention* or program*).ti,* ab*,* kw*,* kf.*

social care/ or (“social care” or “social service*”).ti,* ab*,* kw*,* kf.*

controlled clinical trial/ or equivalence trial/ or pragmatic trial/ or superiority trial/ or non-inferiority trial/ or clinical trial/ or randomized controlled trial/ or adaptive clinical trial/ or “randomized controlled trial (topic)”/ or community trial/ or (trial* or RCT or “randomised control” or “intervention* study” or “experimental study” or “compar* study” or “evaluation study” or “clinical study” or “clinical research” or “prevent* study” or “treatment study”).mp.”

The full search strategies for all other databases are provided in Appendix C, Supplementary Table 3. Advanced search functions, such as Boolean operators, filters, and controlled vocabulary, were used to improve precision and ensure that the search strategies were reproducible. Searches were conducted across the title, abstract and keywords fields in all databases, except for the term “trials”, which included broader fields to account for variations in indexing.

#### Study selection

All references were imported into EndNote for duplicate removal, then into Rayyan for screening. Two reviewers (FK and LS) independently screened titles and abstracts using the inclusion and exclusion criteria, resolving any disagreements through discussion. Full texts were obtained for papers which fulfilled the inclusion criteria, or for those where inclusion based on the abstract alone was unclear. One researcher (FK) conducted full-text screening and recorded reasons for inclusion or exclusion. As data extraction was completed by a single reviewer, all extracted information was verified by a second reviewer to ensure accuracy and consistency.

#### Equity framework for data extraction

To assess how equity was addressed in the included trials, we applied a combined approach using the CORE20PLUS5 and PROGRESS-Plus frameworks (Figs. 1 and 2). CORE20PLUS5 is part of NHS England’s national strategy for reducing health inequalities and is currently in its first year of evaluation [19]. PROGRESS-Plus has been widely applied in assessing equity within health interventions [20] and provides structured definitions of equity-related factors. Published descriptions of the framework were used to justify the inclusion of each factor and to guide data extraction from eligible studies [21–24]. Using these two complementary frameworks provided a consistent basis for identifying, categorising, and extracting data relevant to health inequities and underserved populations, as summarised in Table 2.

**Table 2:** Equity-relevant groups and factors from the combined CORE20PLUS5 and PROGRESS-Plus frameworks with justifications for inclusion

Equity factor	Specific characteristics for extraction	Justification for inclusion(10, 20,)
Place of residence	• Urban/rural location • Housing type	Factors such as green space, air pollution, quality of housing and access to healthcare services are important determinants of health.
Race/ethnicity/culture/language	• Ethnicity/ethnic background • Racial/cultural background • Country of birth • Language spoken	Ethnicity involves shared origin, culture and potentially language and is largely socially defined. Racial health inequalities tend to result from discrimination and systemic disadvantage. Language barriers create communication problems with healthcare providers.
Occupation	• Employment status • Occupation type	Employment and type of occupation are linked to health outcomes and mortality.
Gender/sex	• Gender identity • Sex • Sex assigned at birth	For the purposes of this review, sex refers to biological difference between males and females. This can affect susceptibility to disease and health needs. Gender is socially constructed and affects roles and expectations that can lead to health inequalities.
Religion	• Faith group/religious affiliation	Religious adherence may affect access to necessary care.
Education	• Educational attainment • Literacy/numeracy • School type (public/private)	Influences employment opportunities and income levels. Those with lower education levels are less likely to adopt positive health behaviours and are more likely to access health and social services later.
Socioeconomic status	• SES category • Individual or household income • Receipt of public assistance • IMD (Indices of Multiple Deprivation)	Lower SES and income levels are associated with poorer living conditions, reduced access to nutritious food and worse health and social care outcomes.
Social capital	• Relationship status • Cohabitants • Church/society/memberships • Significant others/social connectedness	Reduced social capital (e.g. quality and strength of social networks, community engagement) is associated with reduced social cohesion and access to support and services.
Plus factors	• Age	Old age and frailty are linked to reduced independence, social isolation and higher burden of health conditions.
	• Disability status/chronic disease	Chronic disability and multiple long-term health conditions result in physical limitations, increased health and social care needs and social isolation.
	• Sexual orientation	May influence health outcomes due to social stigma and increased barriers to social support.
	• Immigrant/residence status	Migrants with uncertain residency status may face fear of deportation, limited eligibility for public benefits, language barriers and lack of culturally appropriate care.
	• Features of relationships (e.g. dependence on caregivers, domestic violence)	Characteristics of a person’s relationships that may affect their health and opportunities.
	• Time-dependent relationships (e.g. chronic illness, long-term employment)	Periods of change during which an individual may be at higher risk of poorer health and social care outcomes.
Core20 (deprivation)	• Most deprived 20% of population (defined by IMD decile)	This population is more likely to experience worse health outcomes and higher levels of unmet social care needs.
PLUS groups	• Inclusion health groups (people experiencing homelessness, gypsy, Roma and traveller communities, drug and alcohol use, vulnerable migrants, sex workers, people in contact with justice systems, victims of modern slavery) • People with learning disabilities • People with autism • People with multiple long-term health conditions • Other groups defined at a local level	Population groups who face multiple additional and overlapping barriers to accessing care and are often underserved in the health and social care system.
5 (clinical areas of focus)	• Maternity • Severe mental illness (bipolar disorder, schizophrenia and other psychoses) [25] • Chronic respiratory disease • Early cancer diagnosis • Hypertension care-finding and optional management and optimal lipid management	Clinical areas with consequences that disproportionately affect underserved and deprived populations and contribute to preventable morbidity and mortality.

### Data extraction

Data from the included studies were extracted using standardised Excel tables that were pilot tested for consistency and completeness. Extracted data were categorised into three main domains:

- Study characteristics – including author(s), year of publication, UK country, setting, study design, intervention type, and the corresponding domain of social care need.
- Participant characteristics – based on equity-relevant factors defined by the CORE20PLUS5 and PROGRESS-Plus frameworks.
- Outcomes – including any analyses of equity-related differential effects, with studies grouped according to intervention type.

#### Quality assessment of studies

The quality of included studies was assessed using the Joanna Briggs Institute (JBI) critical appraisal tools, using the checklist most appropriately applied to the study design [26].

#### Data analysis

Integrative narrative synthesis was used in line with the SWiM (Synthesis Without Meta-analysis) reporting guideline to increase transparency and methodological rigour [27]. The completed SWiM checklist is available in Appendix B Supplementary Table 2. The synthesis focused on how equity was considered in study design, reporting, and analysis with respect to equity-relevant populations and characteristics. A framework was created to classify studies by level of how extensively equity was considered, based on two key criteria, (1) inclusion of equity-relevant populations, as defined by the CORE20PLUS5 and PROGRESS-Plus frameworks and (2) reporting and analytical use of equity-relevant characteristics to assess different intervention effects across subgroups, described in Table 3.

**Table 3:** Equity integration framework for assessing included studies based on CORE20PLUS5 and PROGRESS-Plus criteria

Study level	Criteria
Level 1: High	• Include equity-relevant populations (CORE20PLUS5 groups) as the main target population AND • Measure and report some PROGRESS Plus characteristics AND • Conduct subgroup analyses by at least one PROGRESS-Plus characteristic
Level 2: Moderate	Do not exclusively target equity-relevant populations but do: • Measure and report some PROGRESS Plus characteristics AND • Conduct subgroup analyses by at least one PROGRESS-Plus characteristic
Level 3: Low	• Measure and report some PROGRESS Plus characteristics AND • Target general populations only
Level 4: Minimal/none	• Do not target equity-relevant groups • Do not conduct subgroup analyses • Do not report PROGRESS-Plus characteristics

### Ethics

This study was reviewed and approved by the University of Southampton’s Ethics and Research Governance Online (ERGO II) system. Ethical approval was granted on 24th June 2025 under submission ID 103,794.

## Results

**Fig. 4:**
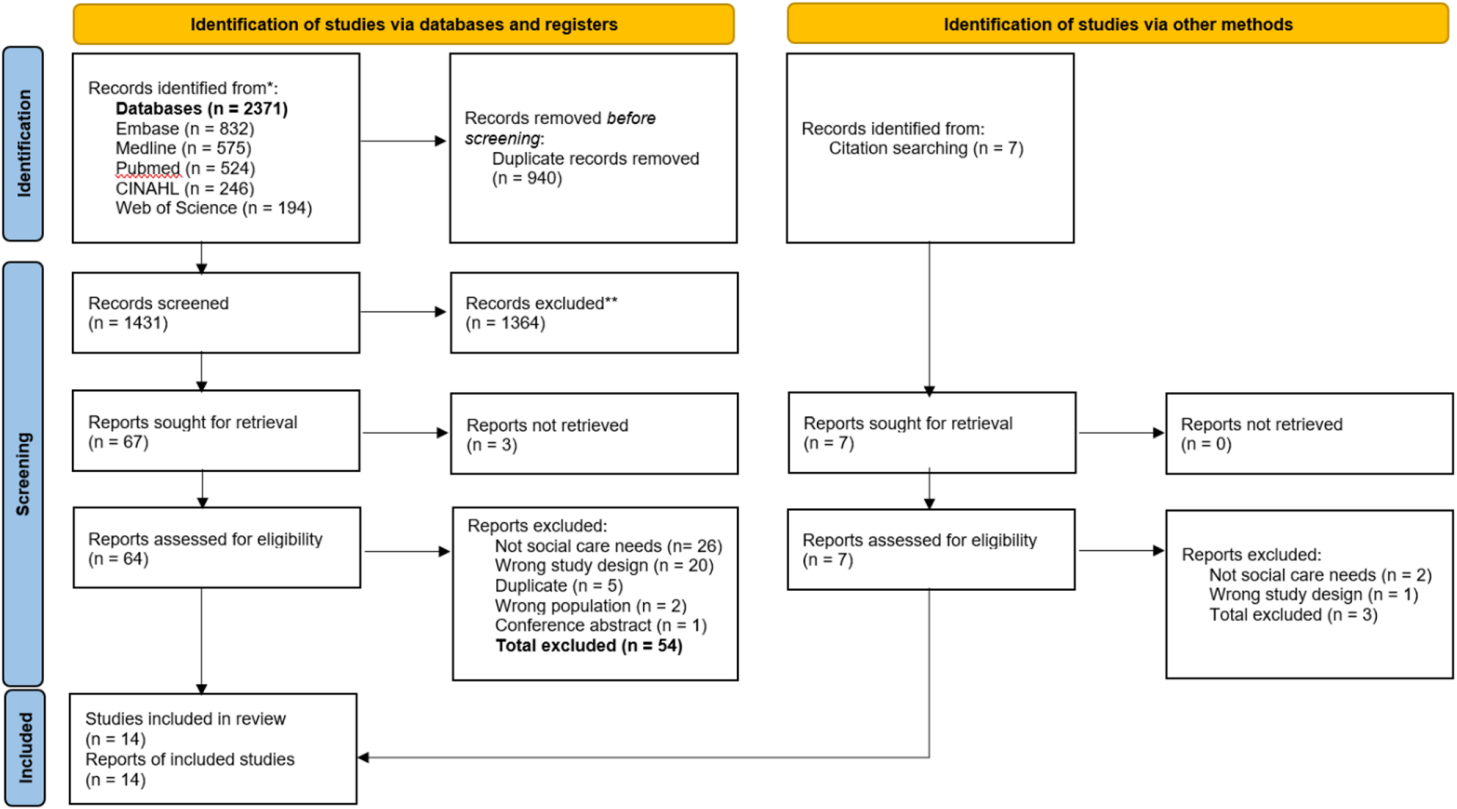
PRISMA flow diagram showing number and source of records identified, screened, excluded and included

The electronic database search found 2,371 records. After removing 940 duplicates, 1431 articles remained. An additional seven records were identified through hand searching of reference lists, producing a total of 1,438 articles to be screened. Title and abstract screening excluded 1,364 articles, leaving 74 for full-text review. Full texts could not be retrieved for three articles. The authors were not contacted for access due to limited time and resource constraints. Of the 71 full texts reviewed, 14 met the inclusion criteria and were included in the final analysis. The most common reasons for exclusion were an ineligible study design and interventions that did not target social care needs. The study selection process is summarised in the PRISMA flowchart in Fig. 4.

### Study characteristics

**Fig. 5:**
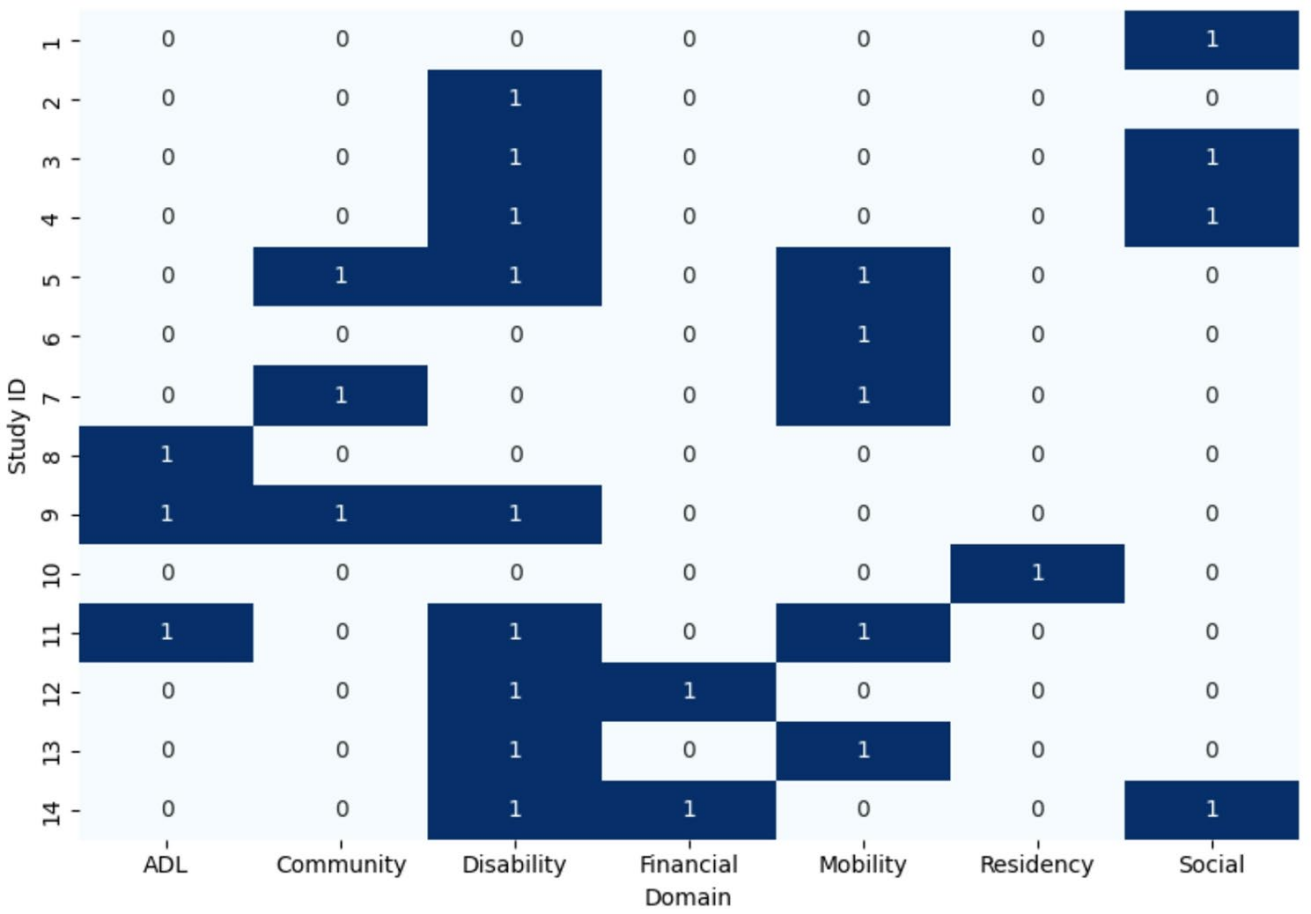
Heat map showing frequency of social care need domains addressed by included studies, where 1 means the domain was present, and 0 means the domain was not present

13 studies were randomised controlled trials, including one feasibility study, and one was a quasi-experimental controlled study. All included studies were conducted in England, with 36% in the South, 29% in the North, and 36% across multiple regions. London was the most common location (*n* = 6). Most studies were community-based (*n* = 12), with additional settings including prisons (*n* = 1) and primary care (*n* = 1). The studies covered diverse populations including adults with specific health conditions (dementia, Parkinson’s disease and hearing loss, *n* = 4), older adults ≥ 65 years (*n* = 3), adults facing financial hardship or living in social housing (*n* = 3), postpartum women (*n* = 1), male prisoners (*n* = 1), adults at risk of loneliness (*n* = 1), and any adults over 60 years (*n* = 1). Most studies (*n* = 10) addressed multiple domains, as shown in Fig. 5. The most common were mobility (*n* = 5), social care networking (*n* = 4) and community care needs (*n* = 4). Activities of Daily Living (ADLs) were recorded in three studies. These refer to the essential self‑care tasks that individuals must be able to perform independently to function safely and effectively in everyday life.

One study focused on residency status, and none focused on bereavement needs.

The included interventions varied but could be grouped into four themes: social support and networking (*n* = 4), health and behaviour change (*n* = 4), integrated or multicomponent care (*n* = 3), and financial and housing support (*n* = 3). Most studies (*n* = 11) used usual care as a comparator. A summary of the characteristics of included studies is presented in Table 4.

### Outcomes and results

Outcomes varied widely, covering mental and physical health, social well-being and quality of life (Table 5). Mental health (*n* = 12) and health-related quality of life (*n* = 9) were the most common. Others included health service use (*n* = 4), social wellbeing (*n* = 3), falls (*n* = 3), physical health (*n* = 3), ADLs (*n* = 3), financial wellbeing (*n* = 2) and time to institutionalisation (*n* = 1). These outcomes were assessed using a wide range of measurement tools. The direction of effect for primary outcome was extracted (as reported by the study authors), using SWiM guidance for narrative synthesis [23]. Six studies reported positive effects, nine no effect, and none reported negative effect, shown in Appendix D Supplementary Table 4.

**Table 4:** Overview of included studies: Key characteristics and intervention details

ID	Authors (Year)	Country (Region)	Study design	Setting	Population	Social care need domain(s) addressed	Intervention	Comparator
Social support and networking								
1	Band et al. (2025) [27]	England (NW (North West), SE (South East)	RCT	Community	Adults at risk of loneliness	Social care networking	Facilitated social networking and reflection	Usual care
2	Coulton et al. (2018) [28]	England (SE)	RCT		Adults ≥ 60 years	Disability	Facilitated singing group	Usual care
3	Stuttard et al. (2021) [29]	England (London)	RCT	Community	Adults ≥ 18 years with severe hearing loss	Disability, Social care and networking	Receipt of trained hearing dog	Usual care
4	Husain et al. (2025) [30]	England (NW, WM (West Midlands), London)	RCT	Community	British South Asian women > 18 within 12 months postpartum	Disability, Social care and networking	CBT-based psychological intervention	Usual care
Health and behaviour change								
5	Bower et al. (2018) [31]	England (NW)	RCT	Community	Adults ≥ 65 years with LTHCs (Long term health conditions) and social care needs	Disability, Community care, Mobility	Telephone health coaching	Usual care
6	Ashburn et al. (2019) [32]	England (SW (South West))	RCT	Prison	Adults with Parkinson’s disease	Mobility	Postural, balance and strengthening exercises supported by physiotherapists and informational DVD	Usual care plus informational DVD and single advice session at trial end
7	Cockayne et al. (2021) [33]	England (North, not specified)	RCT (feasibility study)	Community	Adults ≥ 65 years with a history of falls	Mobility, community care,	Environmental falls risk assessment and modification	Usual care
8	Walters et al.(2017) [34]	England (East, NE (North East)	RCT		Adults ≥ 65 years with mild frailty	ADLs	Goal setting, planning and motivational support for improving and maintaining independence	Usual care
Integrated or multicomponent care								
9	Yilmaz et al. (2024) [35]	England (London, SE, East, Yorkshire and the Humber)	RCT	Primary care	Adults with dementia and their carers	ADLs, Disability, Community care	Multicomponent: behavioural interventions, carer support, psychoeducation, enablement and environmental adaptation	Usual care plus goalsetting
10	Gathercole et al. (2021) [36]	England (Yorkshire and The Humber, East, London, EM (East Midlands), SE)	RCT	Community	Adults with dementia	Residency status	Assessment of needs and full ATT (Assistive technology and telecare) package	Assessment of needs and basic ATT package
11	Forsyth et al. (2021) [37]	England (North, not specified)	RCT	Community	Male prisoners ≥ 50 years	ADLs, Mobility, Disability	Social needs assessment and care plan	Usual care
Financial and housing support								
12	Woodhead et al. (2017) [38]	England (London)	Quasi-experimental controlled study	Primary care	Adults ≥ 18 years requiring welfare advice	Financial, Disability	Advice from welfare advice services located within GP practices	Usual care
13	Cheshire et al. (2018) [39]	England (London)	RCT	Community	Adults ≥ 50 years living in social housing	Disability, Mobility	Two arms: (1) signposting service by social housing manager, (2) intensive handholding service from health and well-being support workers	Usual care
14	Howel et al. (2019) [25]	England (NE)	RCT	Community	Adults > 60 years requiring welfare advice	Financial, Disability, Social care and networking	Domiciliary welfare rights advice, assistance with benefit claims and follow-up as required	Usual care

**Fig. 6:**
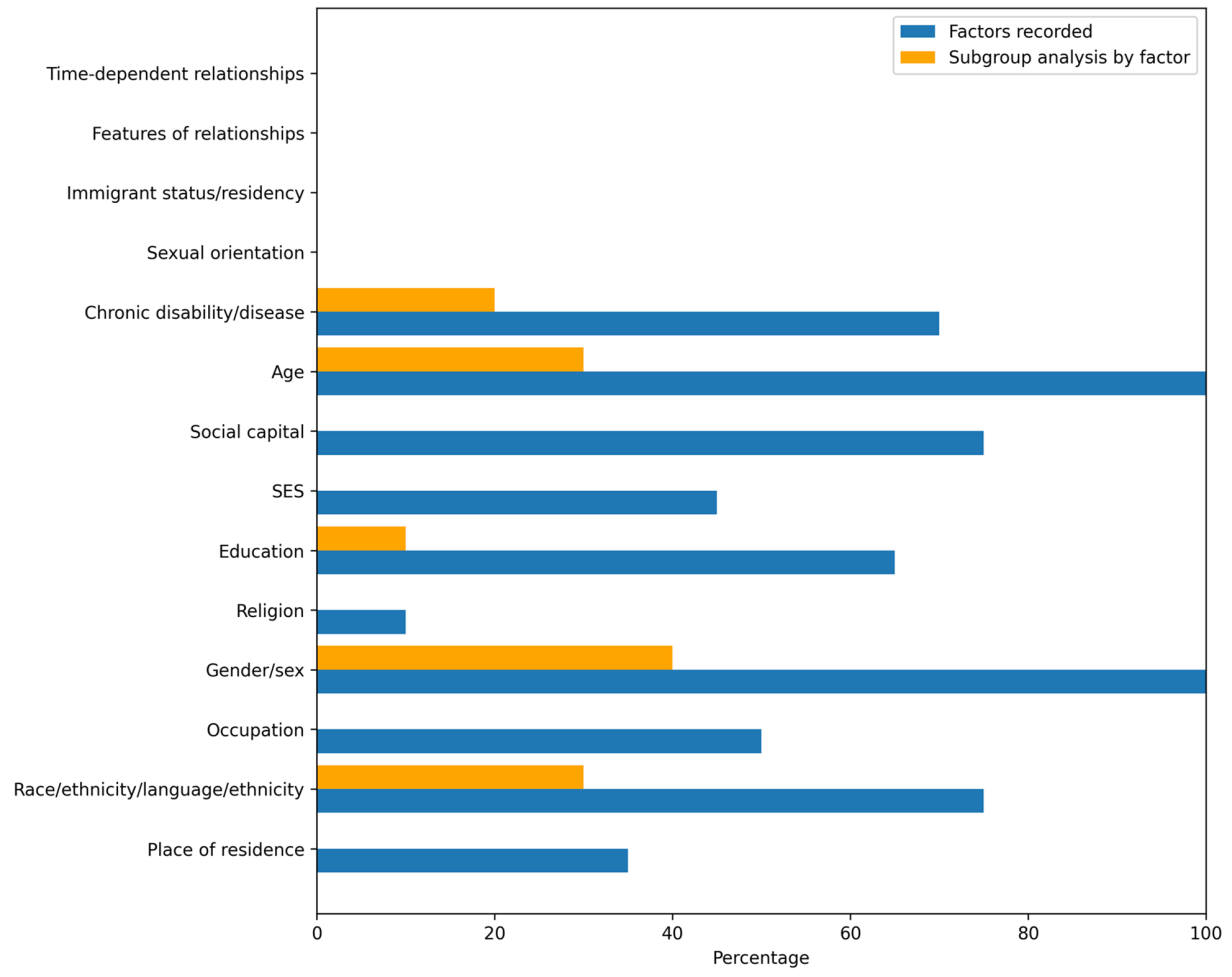
Percentage of included studies reporting each PROGRESS-Plus factor in *any form*

Studies were grouped by level of equity integration: high (*n* = 5), moderate (*n* = 6), and low (*n* = 3). No studies met the criteria for Level 4 (Table 3). Six studies explicitly targeted underserved populations or clinical priority areas defined by CORE20PLUS5, while three others included CORE20PLUS5 groups within their samples, though these typically represented a small proportion of participants. An exception was study ID 13, in which approximately 70% of participants were from ethnic minority backgrounds. Figure 6 presents the proportion of studies recording data or conducting subgroup analyses by equity factor. All studies reported at least one PROGRESS-Plus characteristic (Appendix E Supplementary Table 5). Demographic variables were the most consistently reported, with sex and age included in all studies. In contrast, language and country of birth were rarely captured, reported in only three studies. Five studies undertook subgroup or interaction analyses using PROGRESS-Plus factors; however, these analyses were limited, inconsistent, and often underpowered due to small subgroup sizes. Gender was the most frequently examined factor (*n* = 4) (Appendix F Supplementary Table 6).

**Table 5:**
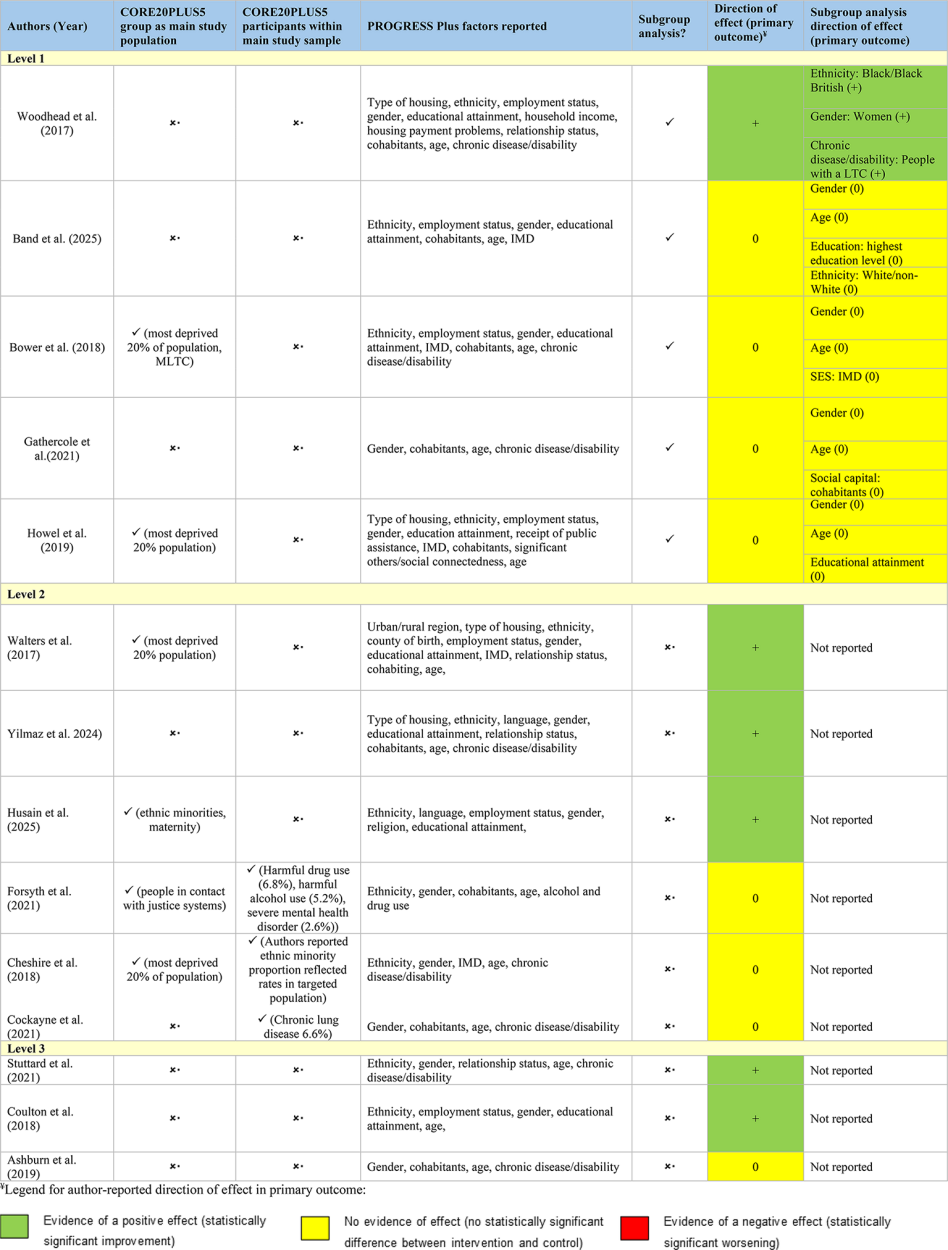
Summary of equity considerations across included studies

**Fig. 7:**
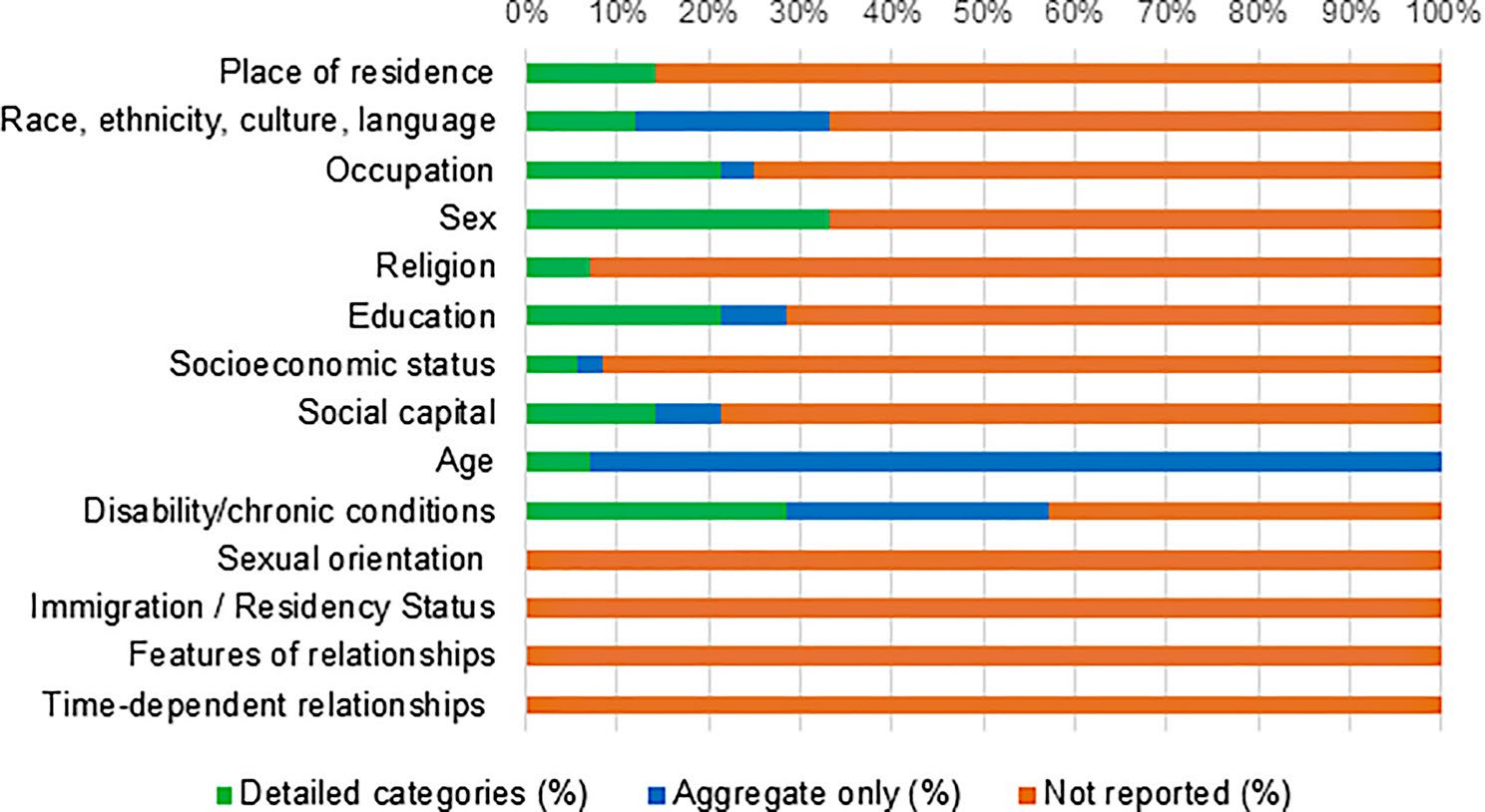
Level of detail in PROGRESS-Plus reporting across studies (disaggregated vs. aggregated data)

Reporting of PROGRESS-Plus factors was inconsistent across studies. Key variables such as ethnicity, income, and housing were often collapsed into broad categories. Ethnicity data were particularly limited, with many studies aggregating diverse populations into single or binary classifications (e.g. “Black” or “Other”). Nearly half of the studies (*n* = 7) reported data on disability or chronic illness; of these, some specified diagnoses while others used broad labels such as “long-term conditions”. Financial information was reported in only two studies, which captured either ability to manage on current income (ID 12) or receipt of public assistance (ID 14), both reduced to dichotomous yes/no responses. By contrast, housing and employment data were generally presented in more detail, with housing status typically disaggregated into categories such as “rented”, “private”, and “owner-occupied”. Across studies, there were also notable omissions. None reported or conducted subgroup analyses on gender identity, sexual orientation, literacy, immigration status, socioeconomic category, features of relationships, or time-dependent relationships, despite these factors being highly relevant to social care needs. Figure 7 summarises the extent to which equity variables were reported in aggregated versus disaggregated form across all studies.

Examination of how studies reported and applied equity relevant characteristics showed substantial variability in both completeness and level of detail. While all studies reported at least one PROGRESSPlus factor, age and sex were the only characteristics captured consistently across all trials, and several important factors - such as sexual orientation, immigration status, literacy, and gender identity - were not reported by any study. Other characteristics, including ethnicity, income, and housing status, were frequently presented in aggregated or simplified forms, limiting the potential for meaningful subgroup analysis. To understand how these characteristics were used analytically, subgroup and interaction analyses were mapped across studies. Only five studies conducted any subgroup analyses, and these were typically limited to gender or age. More complex PROGRESSPlus factors were rarely incorporated into statistical models, as shown in Appendix G Supplementary Table 7. This limited use of equity characteristics in analysis contributed to most studies being classified as moderate or low in equity integration. Direction of effect for each primary outcome was coded using author reported interpretations and SWiM guidance. Six studies demonstrated a positive effect and nine reported no effect, with no study reporting a negative effect. In studies that conducted subgroup analyses, effects were generally consistent across groups, although isolated differences (e.g., gender specific patterns) were noted in a small number of cases. These patterns are summarised in Table 5 and Appendix F Supplementary Table 6. Overall, analysis of reporting patterns, subgroup approaches, and direction of effect data highlights significant variation in how equity considerations were embedded across studies, shaping their classification within the equity integration framework.

### Quality assessment of included studies

Most RCTs met key JBI risk-of-bias criteria, including randomisation, allocation concealment, baseline comparability, treatment consistency, reliable outcome measurement, and complete follow-up [22]. This indicates minimal risk of bias and supports attribution of observed effects to the interventions. All RCTs also scored well on statistical conclusion validity, reinforcing the reliability of findings. Overall, methodological quality was high, providing cautious confidence in the results. Completed checklists are provided in Appendices H – I Supplementary Tables 8,  9 with study-specific assessments in Appendix J Supplementary Table 10.

### Conceptual model

**Fig. 8:**
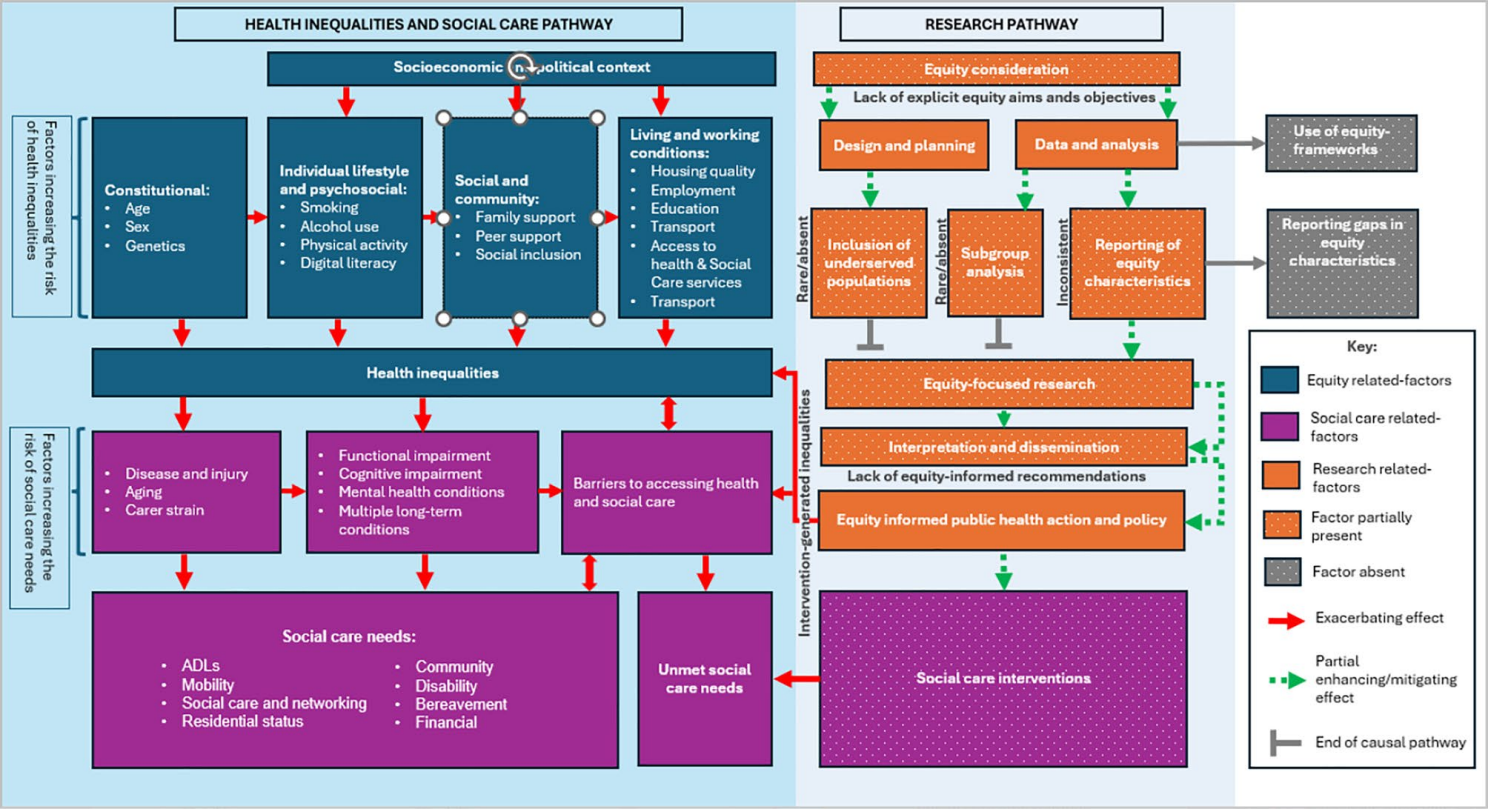
Conceptual model summarising current state of equity integration in social care research based on the findings from this review

Figure 8 presents a conceptual model illustrating how limited equity considerations in social care research contribute to unmet care needs and widening health inequalities. On the research pathway (right side of the model), underserved populations are rarely included, subgroup analyses are infrequent, and equity characteristics are reported inconsistently. Methodological gaps are evident in the limited application of equity frameworks and poor reporting of key variables. Although such studies often perform well on conventional risk-of-bias assessments, they provide limited evidence to inform equitable policy or practice and are insufficient for designing inclusive social care interventions. As shown in the model, inequitable interventions only partially address social care needs and may generate further disparities, creating additional barriers to accessing health and social care.

## Discussion

### Summary

This review synthesised how equity-relevant characteristics, defined by the CORE20PLUS5 and PROGRESS-Plus frameworks, are reported, categorised, and analysed in UK trials of adult social care interventions across the eight social care need domains described by Isaac et al. (2025). Fourteen studies met the inclusion criteria. All reported at least one equity characteristic, but reporting and analysis were inconsistent. Ethnicity was often collapsed into broad categories such as “White”, “Black”, or “Other”, limiting subgroup analysis. Few studies explicitly targeted underserved populations or designed interventions to address inequity, and subgroup analyses were rare and poorly described. Key gaps included gender identity, sexual orientation, immigration status, and literacy. Overall, the findings reveal a disconnect between UK equity policy objectives and the design and reporting of social care research, leaving important blind spots in understanding how interventions affect diverse and disadvantaged populations.

### Comparison to existing literature

Similar to other equity focused systematic reviews, we found that well-designed trials often do not collect or use data on equity-related factors [40], for example, Madani et al. Karran et al., noted demographic data was prioritised while other equity factors were omitted [21, 33], and Nyanchoka et al. found that participant factors such as literacy, sexual orientation and immigration status were rarely or never included in cardiovascular trials [28]. Even when trials met conventional risk-of-bias thresholds, subgroup analyses were often underpowered or omitted entirely, and data on underserved populations was limited, echoing findings from Attwood et al., 2016 [22]. Our findings highlight that researchers should consider equity related characteristics during intervention design and plan subgroup analyses in advance, ensuring the necessary data are collected to support them. While collapsing categories (e.g., combining ethnic groups) can increase sample size, this risks obscuring important differences; broad labels such as “Black” may group populations with distinct cultural and historical backgrounds (e.g., Black Caribbean vs. Black African), leading to misinterpretation of effects [41]. Adequate planning and resourcing are therefore essential to enable meaningful and interpretable equity-focused analyses.

This gap partly reflects limitations in current quality appraisal tools, such as JBI and the Cochrane Risk of Bias 2 (RoB 2) tool, which rarely consider equity-related domains like sample representativeness or differential effects across groups [42]. As highlighted by Abdelmalak et al. (2024), this means trials can score highly on risk-of-bias assessments while contributing little to equity-informed decision-making [43]. As a result, while the evidence base appears internally robust, its external validity, particularly for underserved populations, is uncertain which could widen health inequity, in line with Witham et al. who found that participants recruited into trials often fail to reflect the broader target population that would potentially benefit from the intervention [44]. Research shows that social care expenditure is lower for people from deprivation, especially those who are from ethnic minorities [45] despite these groups being at greater risk of need [46]. A reason for this may be due to the inverse care law, which states that those that need the most access to care may face barriers, such as lack of availability of services, reduced continuity of care, or concerns of trust of stigma in asking for support for social care needs [47]. Consequently, it may be the case that social care need in these populations is underestimated, hence the lack of interventions social care interventions found to target underserved populations in this review.

Another explanation for lack of representation of underserved groups in social care intervention research may be due to difficulty in accessing these populations [12]. NIHR INCLUDE advocates, along with other academic fields outside of health, the need to build inclusive relationships with public contributors from these under-served communities as a key strategy to increase inclusion, through co-production methods, such as participatory research [48]. However, it has been noted that researchers do not always have the knowledge on the best methods for building trustful relationships [49] and other practical barriers such as the time taken to build sustainable relationships and resources available can undermine this process, resulting in under-served individuals feeling as though their involvement in the research process is a ‘box ticking’ exercise [50] These are key issues which need to be successfully address to incorporate wider participation from underrepresented groups, to reduce health inequity within social care research, and to avoid further intervention generated inequity [41].

#### Strengths and limitations

To our knowledge, this is the only systematic review applying an equity lens to adult social care interventions. It adds to the current body of evidence by demonstrating that challenges already identified in health-equity research, such as inconsistent reporting, minimal subgroup analysis and lack of standardisation, are also present in the social care sector. Notably, it also adds new evidence by highlighting the widespread use of aggregated equity categories within primary studies, which limits meaningful analysis of intervention effects. By highlighting gaps between policy aims and how research is being conducted, this review provides timely insights to support more equitable decision-making. This review used robust and transparent methods, following PRISMA guidance. The systematic search strategy was inclusive and covered a range of relevant databases. Hand-searching of references and using a combination of MeSH and free text terms improved inclusivity. By focusing the search on the last 10 years, it ensured the review reflects current social care policies, frameworks and practices in the UK.

However, restricting the review to English-language and UK-based studies may have excluded relevant evidence for migrant or ethnic-minority groups and limited generalisability beyond the UK, additionally, excluding grey literature may have overlooked emerging findings. The equity assessment tool developed has not been externally validated, reducing comparability with other reviews. While PROGRESS-Plus and CORE20PLUS5 frameworks are widely used, they have limitations, including potential omission of context-specific factors like digital exclusion and so findings should be interpreted with caution, recognising that overlapping or local disadvantages may not be fully captured. Full‑text screening was conducted by a single reviewer, which may introduce a risk of selection bias. However, to mitigate this, any studies where relevance was uncertain were discussed collaboratively with a second reviewer (LS) to reach consensus. While this approach does not fully eliminate the limitations associated with single‑reviewer screening, it reduces the likelihood of erroneous exclusions and enhances the reliability of the screening process.

#### Implications

**Fig. 9:**
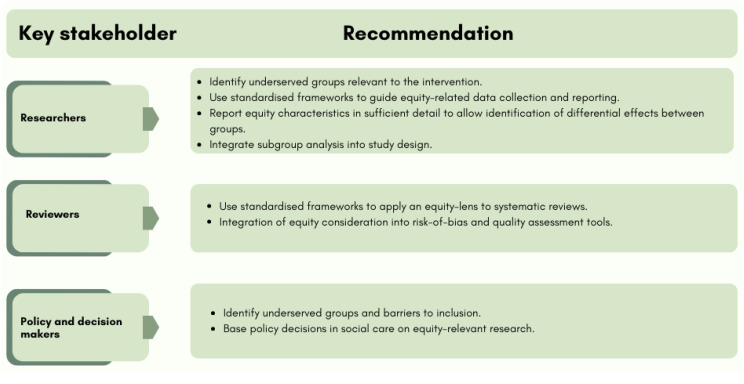
Recommended actions for key stakeholders in social care research and policy based on summary of findings from a systematic review examining equity of UK trials for social care interventions

This review highlights a disconnect between the aims of UK health and social care policy, which emphasise reducing inequity, and the way research is conducted. Evidence often assumes interventions are equally effective across populations, despite limited consideration of underserved groups. With rising demand for adult social care and widening inequalities, producing equity-relevant research is essential to ensure interventions benefit all populations. To address this, studies should identify underserved groups and adopt inclusive recruitment strategies, supported by frameworks such as PROGRESS-Plus to standardise equity-related data collection and reporting. Integrating equity into study design, analysis, and quality assessment will enhance the relevance and generalisability of findings. Subgroup analyses and the use of standardised reporting categories (e.g. ONS classifications) are critical for detecting differential impacts and informing equitable policy decisions, as illustrated in Fig. 9. Ensuring adequately powered subgroup analyses may necessitate expanded funding and infrastructure support. System-level changes - such as improved routine collection of high-quality equity-relevant data (including clearer distinction between race and ethnicity), and funders prioritising equity considerations in trial design - would help ensure that future research can examine subgroup effects in a robust and interpretable way. Implementing these approaches will help ensure that social care research generates evidence to support social care interventions that are effective and equitable.

## Supplementary Information

Below is the link to the electronic supplementary material.

Supplementary Material 1

## Data Availability

No datasets were generated or analysed during the current study.

## Abbreviations

NW: North-West (England)
OHSCAP: Older prisoner Health and Social Care Assessment and Plan
ONS: Office for National Statistics
PAM: Patient Activation Measure
PASE: Physical Activity Scale for the Elderly
PHQ-9: Patient Health Questionnaire-9
PND: Post-natal depression
PRISMA: Preferred Reporting Items for Systematic Reviews and Meta-Analyses
PROSPERO: International Prospective Register of Systematic Reviews
QOL: Quality of life
QOL-AD: Quality of Life in Alzheimer's Disease
RoB2: Risk of Bias 2
SDSCA: Summary of Diabetes Self-Care Activities
SE: South-East (England)
SF-12: 12 Item Short-Form Survey
SPS: Social Provisions Scale
SW: South-West (England)
SWEMWBS: Short Warwick–Edinburgh Mental Well-Being scale
UK: United Kingdom
WHOQOL-BREF: World Health Organization Quality of Life- Brief Version
WM: West Midlands (England)
WSAS: Work and Social Adjustment Scale
ZBI: 22-item short version of the Zarit Burden Interview
