# Supplementary material for: Equity and inclusion in UK adult social care: a systematic review of trials
Source: BMC Health Serv Res. 2026 Mar 17;26:586. doi: 10.1186/s12913-026-14347-y (PMC13112718; doi:10.1186/s12913-026-14347-y)
Supplement: Supplementary file 1 — Supplementary Material 1 [file 12913_2026_14347_MOESM1_ESM.docx]

1. **Appendices**

Appendix A: Supplementary Table 1: Domains and definition of social care needs (taken directly from Issac et al.(12)

| **Domain** | **Description** |
| --- | --- |
| **Activities of daily living needs** | - Social needs associated with carrying out activities of daily living (ADLs) including: - Ambulating - the extent of an individual’s capability to move from one position to another, perform basic movements and dexterity. - Feeding - ability to feed oneself without assistance. - Dressing - ability to put on their clothes and select appropriate clothing. - Personal hygiene - ability to undertake basic washing, bathing, and grooming, maintain personal hygiene including dental, nail, and hair care. - Continence – ability to control bladder and/or bowel function. - Toileting - ability to get to and from the toilet, using it appropriately and cleaning oneself. |
| **Mobility needs** | - Social needs resulting from difficulties with physical mobility, require the provision of mobility aids or equipment, professional input and care and financial support with mobility including: - Wheelchair use. - Non-wheelchair mobility aid use (e.g., walking stick, Zimmer frame, mobility scooter). - Professional care staff provision to assist with mobility. - Receives disability living allowance. - Receives mobility allowance. |
| **Financial needs** | - Social needs relating to challenges an individual may have managing their personal finances employment-related difficulties and receiving assistance from a range of financial support services, including: - Difficulty budgeting and handling money. - Employment status (e.g., unemployed, or long-term sick). - Referral and/or input from financial adviser or financial services. - Receipt of assistance from support services such as food banks, accessing the affordable warm programme. - Difficulty writing cheques, using a credit card, carrying out arithmetic reasoning related to money and difficulty managing bank accounts. |
| **Disability needs** | - Social needs resulting from specific disabilities or impairments including: - Mental health disability. - Intellectual disability. - Learning disability. - Speech, hearing, and sensory disabilities. - Any state assessment of disability. |
| **Community care needs** | - Social needs requiring support from a range of community health and social care services and practitioners, including: - Community physiotherapist or occupational therapy. - Drug and alcohol team. - Mental health team. - Social care services. - Outreach or voluntary services. - Community nurses, specialist nurses or matrons. - Community secondary care professionals. - Audiology. - Palliative care team. - Dietician. - Community care navigator or social care prescribers. - Pharmacy input. |
| **Residency status needs** | - Social needs relating to residential status including: - Hospice care. - Nursing home care. - Care home. - Own home with adaptations. |
| **Social care networking needs** | - Social care needs associated with an individual’s ability to socially connect, network, and participate in social care activities including: - Ability to maintain meaningful relationships with family, friends, and others. - Ability to socialise and mix with others in social care contexts. - Ability to effectively communicate verbally and non-verbally. - Ability to participate in hobbies, leisure, and community activities. - Access to and ability to organise transportation. - Input from professional services to socially connect and participate such as day units or visits from charities for loneliness. |
| **Bereavement needs** | - Social care needs arising from family bereavement including: - Death of a partner, husband, wife, sibling, child including neonatal and postnatal death, sudden infant death, maternal death. - Input from professional services including referral to or use of bereavement counselling or therapies. |

Appendix C: Supplementary Table 2: Search terms used by database

| **Embase (OVID).** Extraction date: 14^th^ June 2025 | | |
| --- | --- | --- |
| **Search #** | **Search terms** | **Output** |
| 1 | adult/ or (adult* or "working age" or older or elderly).ti,ab,kw,kf. | 12,622,935 |
| 2 | United Kingdom/ or (UK or "United Kingdom" or Britain or Great Britain or England or English or Scotland or Scottish or Wales or Welsh or "Northern Ireland" or "Northern Irish").ti,ab,kw,kf. | 975023 |
| 3 | (intervention* or program*).ti,ab,kw,kf. | 3,699,888 |
| 4 | social care/ or ("social care" or "social service*").ti,ab,kw,kf. | 38,995 |
| 5 | controlled clinical trial/ or equivalence trial/ or pragmatic trial/ or superiority trial/ or non-inferiority trial/ or clinical trial/ or randomized controlled trial/ or adaptive clinical trial/ or "randomized controlled trial (topic)"/ or community trial/ or (trial* or RCT or "randomised control" or "intervention* study" or "experimental study" or "compar* study" or "evaluation study" or "clinical study" or "clinical research" or "prevent* study" or "treatment study").mp. | 10,236,966 |
| 6 | 1 and 2 and 3 and 4 and 5 | 865 |
| 7 | limit 6 to English language | 863 |
| 8 | 7 and 2015:2025.(sa_year). | 832 |

| **CINAHL (EBSCOhost)**. Extraction date: 14^th^ June 2025 | | |
| --- | --- | --- |
| **Search #** | **Search terms** | **Output** |
| 1 | (MH "Adult") or XB (adult* or "working age" or older or elderly) | 1,906,001 |
| 2 | (MH "United Kingdom") OR XB (UK or "United Kingdom" or Britain or Great Britain or England or English or Scotland or Scottish or Wales or Welsh or "Northern Ireland" or "Northern Irish") OR MW (UK or "United Kingdom" or Britain or Great Britain or England or English or Scotland or Scottish or Wales or Welsh or "Northern Ireland" or "Northern Irish") OR MH (UK or "United Kingdom" or Britain or Great Britain or England or English or Scotland or Scottish or Wales or Welsh or "Northern Ireland" or "Northern Irish") | 482,418 |
| 3 | XB (intervention* or program*) OR MW (intervention* or program*) OR MH (intervention* or program*) | 1,154,231 |
| 4 | XB ("social care" or "social service*") OR MW ("social care" or "social service*") OR MH ("social care" or "social service*") | 18,896 |
| 5 | (MH "Randomized Controlled Trials") OR (MH "Clinical Trials") OR TX (trial* or RCT or "randomised control" or "intervention* study" or "experimental study" or "compar* study" or "evaluation study" or "clinical study" or "clinical research" or "prevent* study" or "treatment study") | 1,420,480 |
| 6 | 1 AND 2 AND 3 AND 4 AND 5 | 283 |
| 7 | Narrow by Language: English | 281 |
| 8 | Publication date:20150101-20251231 | 246 |

| **PubMed (U.S. National Library of Medicine (NLM).** Extraction date: 14^th^ June 2025 | | |
| --- | --- | --- |
| **Search #** | **Search terms** | **Output** |
| 1 | "adult"[MeSH Terms] OR adult*[Title/Abstract] OR "working age"[Title/Abstract] OR older[Title/Abstract] OR elderly[Title/Abstract] | 950,803 |
| 2 | "United Kingdom"[MeSH Terms] OR  UK[Title/Abstract] OR "United Kingdom"[Title/Abstract] OR  Britain[Title/Abstract] OR "Great Britain"[Title/Abstract] OR England[Title/Abstract] OR English[Title/Abstract] OR Scotland[Title/Abstract] OR Scottish[Title/Abstract] OR Wales[Title/Abstract] OR Welsh[Title/Abstract] OR "Northern Ireland"[Title/Abstract] OR "Northern Irish"[Title/Abstract] | 731,832 |
| 3 | Intervention*[Title/Abstract] OR program*[Title/Abstract] | 262,334 |
| 4 | "Social Work"[MeSH Terms] OR "Social Welfare"[MeSH Terms] OR "social care"[Title/Abstract] OR "social service*"[Title/Abstract] | 9,866 |
| 5 | "Randomized Controlled Trial"[Publication Type] OR  "Clinical Trial"[Publication Type] OR trial*[All Fields] OR RCT[All Fields] OR "randomised control"[All Fields] OR "intervention* study"[All Fields] OR "experimental study"[All Fields] OR "compar* study"[All Fields] OR "evaluation study"[All Fields] OR "clinical study"[All Fields] OR "clinical research"[All Fields] OR "prevent* study"[All Fields] OR "treatment study"[All Fields] | 462,741 |
| 6 | 1 AND 2 AND 3 AND 4 AND 5 | 566 |
| 7 | Filters applied: English | 565 |
| 8 | Filters applied: 10 years | 524 |

| **Web of Science (Clarivate)**. Extraction date: 14^th^ June 2025 | | |
| --- | --- | --- |
| **Search #** | **Search terms** | **Output** |
| 1 | TS=(adult* or "working age" or older or elderly) | 4,633,466 |
| 2 | TS=("United Kingdom" OR UK OR Britain OR "Great Britain" OR England OR Scotland OR Wales OR "Northern Ireland") | 784,750 |
| 3 | TS=(intervention* or program*) | 4,534,070 |
| 4 | TS=("social care" or "social service*") | 39,223 |
| 5 | ALL=(trial* or RCT or "randomised control" or "intervention* study" or "experimental study" or "compar* study" or "evaluation study" or "clinical study" or "clinical research" or "prevent* study" or "treatment study") | 3,221,128 |
| 6 | #1 AND #2 AND #3 AND #4 AND #5 | 286 |
| 7 | #5 AND #4 AND #3 AND #2 AND #1 and English (Languages) | 286 |
| 8 | #1 AND #2 AND #3 AND #4 AND #5 and English (Languages) and 2025 or 2024 or 2023 or 2022 or 2021 or 2020 or 2019 or 2018 or 2017 or 2016 or 2015 (Publication Years) | 194 |

Appendix D: Supplementary Table Four, Summary of outcomes, measurement tools and author-reported conclusions of included studies

| ID | Authors (Year) | Outcome(s) | Measurement tools | Direction of effect (primary outcome)^¥^ | Author-reported key conclusions |
| --- | --- | --- | --- | --- | --- |
| Social support and networking | | | | | |
| 1 | Band et al. (2025) | Mental wellbeing (primary), physical health, loneliness | SF-12 (12 Item Short-Form Survey),, DJGS (De Jong Gierveld Scale), Campaign Against Loneliness tool, DSSI (Duke Social Support Index), SWEMWBS (Short Warwick–Edinburgh Mental Well-Being scale), CENS (Collective Efficacy Network Scale), SPS (Social Provisions Scale), Brief IPQ (Brief Illness Perception Questionnaire Brief) | 0 | No evidence of a significant impact on mental health, physical health or other outcomes (including loneliness and isolation) |
| 2 | Coulton et al. (2018) | Mental wellbeing (primary), health-related QOL (quality of life), anxiety | SF-12, HADS (Hospital Anxiety and Depression Scale) | + | Community group singing was associated with significant improvements in mental health-related quality of life, anxiety and depression, suggesting it may be a useful intervention to support older adults’ mental health |
| 3 | Stuttard et al. (2021) | Mental wellbeing (primary), social functioning, anxiety, perceived dependency | SWEMWBS, WSAS (Work and Social Adjustment Scale), GAD-7 (Generalized Anxiety Disorder 7-item scale), PHQ-9 (Patient Health Questionnaire-9), Hearing Loss Questionnaire, self-rating of dependency | + | Short-term, statistically significant improvements in wellbeing, mental health, functioning, social isolation, and dependency for hearing dog recipients |
| 4 | Husain et al. (2025) | Depression | HDRS (Hamilton Depression Rating Scale) | + | Strong evidence that a culturally tailored group CBT intervention accelerates recovery from postnatal depression in British South Asian women within the early months postpartum (although effects diminish by one year) |
| Health and behaviour change | | | | | |
| 5 | Bower et al. (2018) | Health-related QOL (primary), confidence to manage own healthcare, quality of life (primary), depression, diabetes self-care, health service utilisation | EQ-5D-5L ((EuroQol five-dimension five-level questionnaire), PAM (Patient Activation Measure), WHOQOL-BREF (World Health Organization Quality of Life- Brief Version), MHI-5 (Mental Health Inventory-5), SDSCA (Summary of Diabetes Self-Care Activities) | 0 | Telephone health coaching alongside existing services did not significantly improve health-related quality of life in older adults with long-term health conditions and social care needs |
| 6 | Ashburn et al. (2019) | Falls (primary), near falls, strength, falls efficacy, freezing of gait, depression, physical activity, health-related QOL | Self/carer-reported falls, Mini-BESTtest, Chair Stand Test, FoG (Freezing of Gait Scale), GDS (Geriatric Depression Scale), PASE (Physical Activity Scale for the Elderly) | 0 | No significant reduction in repeat falls, but improvements were noted in balance, strength, falls efficacy, and reduced near-falls |
| 7 | Cockayne et al. (2021) | Falls (primary), fracture rate, fear of falling, health-related QOL, health service utilisation | Self-reported falls, fear of falling and health service utilisation, EQ-5D-5L | 0 | There was no effect on the rate of self-reported falls among older adults at increased risk of falling |
| 8 | Walters et al. (2017) | Feasibility (primary), ADLs, grip strength, gait speed, physical activity, mental wellbeing, general health, health-related QOL, falls, smoking and alcohol use (primary clinical outcome not specified) | MBI (Modified Barthel Index), IPAQ-E (International Physical Activity Questionnaire modified), WEMWBS, GHQ-12 (The General Health Questionnaire-12), EQ-5D-5L, AUDIT-C (Alcohol Use Disorders Identification Test- Shortened version) | + | There were significant improvements ADLs, grip strength and psychological distress. There were no significant differences in any other outcome. The small-scale feasibility trial indicated that the intervention/service would be well-received and can be delivered at a modest cost |
| Integrated or multicomponent care | | | | | |
| 9 | Yilmaz et al. 2024) | Carer-reported achievement of personalised goals (primary), ADLs, health-related QOL, service and support utilisation, time until care home admission or death, carer anxiety/depression | GAS (Carer-rated Goal Attainment Scaling), DAD (Disability Assessment for Dementia scale), DEMQOL/DEMQOL-proxy (Dementia Quality of Life instrument-proxy), Client Service Receipt Inventory, Hospital Anxiety and Depression Scale | + | The intervention improved personalised attainment of personalised goals over two years |
| 10 | Gathercole et al.(2021) | Time to institutionalisation, (primary), health-related QOL, carer burden, healthcare utilisation | Carer-reported/health records, QOL-AD | 0 | The full ATT package did not significantly extend the time people with dementia remained in the community, nor did it reduce caregiver burden, depression, or anxiety |
| 11 | Forsyth et al. (2021) | Mean number of unmet needs assessed (primary), specific social care need domains, depressive symptoms, health-related QOL, ADLs | CANFOR-SF (Short version of the Camberwell Assessment of Need: Forensic Version), OHSCAP (Older prisoner Health and Social Care Assessment and Plan), GDS, EQ‑5D‑5L, BADLs (Bristol Activities of Daily Living Scale) | 0 | The intervention did not significantly reduce unmet health and social care needs among older prisoners, nor did it improve quality of life, daily functioning, or depressive symptoms |
| Financial and housing support | | | | | |
| 12 | Woodhead et al. (2017) | Mental health (primary), mental wellbeing, financial strain and support seeking, healthcare usage | GHQ-12, SWEMWBS, financial strain self-reported, GP consultation rate | + | Co-located welfare advice improves short-term mental health and well-being, reduces financial strain and delivers substantial financial benefits |
| 13 | Cheshire et al. (2018) | General health (primary), mental wellbeing, health behaviours (e.g. smoking levels and alcohol intake) | Self-reported health, loneliness, activity, mobility and health behaviours, ONS (Office for National Statistics) wellbeing measure, SWEMWBS | 0 | These interventions for a targeted group led to significant reductions in NHS usage, along with other health benefits that were not statistically significant |
| 14 | Howel et al. (2019) | Health-related QOL (primary), mental wellbeing, financial well-being, standard of living, social support and participation, general health, health related behaviours (e.g. smoking), physical activity | CASP-19 (Control, Autonomy, Self-realisation and Pleasure 19 Score), PHQ-9, self-reported financial wellbeing, standard of living, social support and participation and health-related behaviours, EQ-5D-3L, PASE | 0 | There was no evidence to support domiciliary welfare rights advice as a means of promoting health among older people. |

^¥^Legend for author-reported direction of effect in primary outcome:


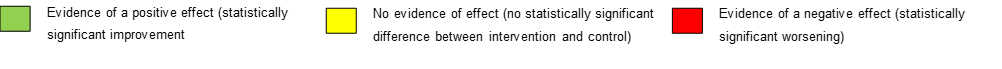


Appendix E: Supplementary Table 5: Frequency of PROGRESS Plus characteristics recorded by study

| **Study ID** (Year) | **Place of residence** | **Race/ethnicity/language** | **Occupation** | **Gender/sex** | **Religion** | **Education** | **SES** | **Social capital** | **Age** | **Chronic disability/disease** | **Sexual orientation** | **Immigrant status/residency** | **Features of relationships** | **Time-dependent relationships** | **Percentage of factors factors included^¥^** |
| --- | --- | --- | --- | --- | --- | --- | --- | --- | --- | --- | --- | --- | --- | --- | --- |
| Band et al. (2025) | ✓ | ✓ | ✓ | ✓ | 🗶 | ✓ | 🗶 | ✓ | ✓ | 🗶 | 🗶 | 🗶 | 🗶 | 🗶 | 50% |
| Bower et al. (2018) | 🗶 | ✓ | ✓ | ✓ | 🗶 | ✓ | ✓ | ✓ | ✓ | ✓ | 🗶 | 🗶 | 🗶 | 🗶 | 57% |
| Stuttard et al. (2021) | 🗶 | ✓ | 🗶 | ✓ | 🗶 | 🗶 | 🗶 | ✓ | ✓ | ✓ | 🗶 | 🗶 | 🗶 | 🗶 | 36% |
| Woodhead et al. (2017) | ✓ | ✓ | ✓ | ✓ | 🗶 | ✓ | ✓ | ✓ | ✓ | ✓ | 🗶 | 🗶 | 🗶 | 🗶 | 64% |
| Yilmaz et al. 2024) | ✓ | ✓ | 🗶 | ✓ | 🗶 | ✓ | 🗶 | ✓ | ✓ | ✓ | 🗶 | 🗶 | 🗶 | 🗶 | 50% |
| Cheshire et al. (2018) | 🗶 | ✓ | 🗶 | ✓ | 🗶 | 🗶 | ✓ | 🗶 | ✓ | ✓ | 🗶 | 🗶 | 🗶 | 🗶 | 36% |
| Gathercole et al.(2021) | 🗶 | 🗶 | 🗶 | ✓ | 🗶 | 🗶 | 🗶 | ✓ | ✓ | ✓ | 🗶 | 🗶 | 🗶 | 🗶 | 29% |
| Forsyth et al.  (2021) | 🗶 | ✓ | 🗶 | ✓ | 🗶 | 🗶 | 🗶 | ✓ | ✓ | 🗶 | 🗶 | 🗶 | 🗶 | 🗶 | 29% |
| Walters et al.  (2017) | ✓ | ✓ | ✓ | ✓ | 🗶 | ✓ | ✓ | ✓ | ✓ | 🗶 | 🗶 | 🗶 | 🗶 | 🗶 | 57% |
| Cockayne et al.  (2021) | 🗶 | 🗶 | 🗶 | ✓ | 🗶 | 🗶 | 🗶 | ✓ | ✓ | ✓ | 🗶 | 🗶 | 🗶 | 🗶 | 29% |
| Coulton et al.  (2018) | ✓ | ✓ | ✓ | ✓ | 🗶 | ✓ | 🗶 | 🗶 | ✓ | 🗶 | 🗶 | 🗶 | 🗶 | 🗶 | 43% |
| Ashburn et al.  (2019) | 🗶 | 🗶 | 🗶 | ✓ | 🗶 | 🗶 | 🗶 | ✓ | ✓ | ✓ | 🗶 | 🗶 | 🗶 | 🗶 | 29% |
| Howel et al.  (2019) | ✓ | ✓ | ✓ | ✓ | 🗶 | ✓ | ✓ | ✓ | ✓ | 🗶 | 🗶 | 🗶 | 🗶 | 🗶 | 57% |
| Husain et al.  (2025) | 🗶 | ✓ | ✓ | ✓ | ✓ | ✓ | 🗶 | 🗶 | ✓ | 🗶 | 🗶 | 🗶 | 🗶 | 🗶 | 43% |
| **Percentage of studies including factor^¥^** | 36% | 71% | 50% | 100% | 7% | 57% | 36% | 79% | 100% | 57% | 0% | 0% | 0% | 0% |  |

¥ Legend for frequency of reporting of PROGRESS Plus characteristics:

| **Frequency of reporting** | **Range** |
| --- | --- |
| Commonly | 76-100% |
| Often | 51-75% |
| Sometimes | 26-50% |
| Rarely | 0-25% |

Appendix F**:** Supplementary Table 6: Subgroup analysis of PROGRESS Plus characteristics by study

| **Study ID** (Year) | **Place of residence** | **Race/ethnicity/language** | **Occupation** | **Gender/sex** | **Religion** | **Education** | **SES** | **Social capital** | **Age** | **Chronic disability/disease** | **Sexual orientation** | **Immigrant status/residency** | **Features of relationships** | **Time-dependent relationships** | **Percentage of factors factors included^¥^** |
| --- | --- | --- | --- | --- | --- | --- | --- | --- | --- | --- | --- | --- | --- | --- | --- |
| Band et al. (2025) | 🗶 | ✓ | 🗶 | ✓ | 🗶 | ✓ | 🗶 | 🗶 | ✓ | 🗶 | 🗶 | 🗶 | 🗶 | 🗶 | 29% |
| Bower et al. (2018) | 🗶 | 🗶 | 🗶 | ✓ | 🗶 | 🗶 | ✓ | 🗶 | ✓ | ✓ | 🗶 | 🗶 | 🗶 | 🗶 | 29% |
| Stuttard et al. (2021) | 🗶 | 🗶 | 🗶 | 🗶 | 🗶 | 🗶 | 🗶 | 🗶 | 🗶 | 🗶 | 🗶 | 🗶 | 🗶 | 🗶 | 0% |
| Woodhead et al. (2017) | 🗶 | ✓ | 🗶 | ✓ | 🗶 | 🗶 | 🗶 | 🗶 | 🗶 | 🗶 | 🗶 | 🗶 | 🗶 | 🗶 | 14% |
| Yilmaz et al. 2024) | 🗶 | 🗶 | 🗶 | 🗶 | 🗶 | 🗶 | 🗶 | 🗶 | 🗶 | 🗶 | 🗶 | 🗶 | 🗶 | 🗶 | 0% |
| Cheshire et al. (2018) | 🗶 | 🗶 | 🗶 | 🗶 | 🗶 | 🗶 | 🗶 | 🗶 | 🗶 | 🗶 | 🗶 | 🗶 | 🗶 | 🗶 | 0% |
| Gathercole et al.(2021) | 🗶 | ✓ | 🗶 | ✓ | 🗶 | 🗶 | 🗶 | ✓ | ✓ | 🗶 | 🗶 | 🗶 | 🗶 | 🗶 | 29% |
| Forsyth et al.  (2021) | 🗶 | 🗶 | 🗶 | 🗶 | 🗶 | 🗶 | 🗶 | 🗶 | 🗶 | 🗶 | 🗶 | 🗶 | 🗶 | 🗶 | 0% |
| Walters et al.  (2017) | 🗶 | 🗶 | 🗶 | 🗶 | 🗶 | 🗶 | 🗶 | 🗶 | 🗶 | 🗶 | 🗶 | 🗶 | 🗶 | 🗶 | 0% |
| Cockayne et al.  (2021) | 🗶 | 🗶 | 🗶 | 🗶 | 🗶 | 🗶 | 🗶 | 🗶 | 🗶 | 🗶 | 🗶 | 🗶 | 🗶 | 🗶 | 0% |
| Coulton et al.  (2018) | 🗶 | 🗶 | 🗶 | 🗶 | 🗶 | 🗶 | 🗶 | 🗶 | 🗶 | 🗶 | 🗶 | 🗶 | 🗶 | 🗶 | 0% |
| Ashburn et al.  (2019) | 🗶 | 🗶 | 🗶 | 🗶 | 🗶 | 🗶 | 🗶 | 🗶 | 🗶 | 🗶 | 🗶 | 🗶 | 🗶 | 🗶 | 0% |
| Howel et al.  (2019) | 🗶 | 🗶 | 🗶 | 🗶 | 🗶 | 🗶 | 🗶 | 🗶 | ✓ | 🗶 | 🗶 | 🗶 | 🗶 | 🗶 | 7% |
| Husain et al.  (2025) | 🗶 | 🗶 | 🗶 | 🗶 | 🗶 | 🗶 | 🗶 | 🗶 | 🗶 | 🗶 | 🗶 | 🗶 | 🗶 | 🗶 | 0% |
| **Percentage of studies including factor^¥^** | 0% | 21% | 0% | 29% | 0% | 7% | 7% | 7% | 21% | 7% | 0% | 0% | 0% | 0% |  |

¥ Legend for frequency of subgroup analysis of PROGRESS Plus characteristics:

| **Frequency of included subgroup analysis** | **Range** |
| --- | --- |
| Commonly | 76-100% |
| Often | 51-75% |
| Sometimes | 26-50% |
| Rarely | 0-25% |

Appendix G: Supplementary Table 7: Level of detail of PROGRESS-Plus characteristics reported by included studies

| **PROGRESS-Plus factor** | **Characteristic** | **Detail level (% of participants with characteristic included)** |
| --- | --- | --- |
| **Place of residence** | **Urban/rural** | |
|  | Walters et al. | Urban (49%), semi-urban (51%) |
|  | **Type of housing** | |
|  | Howel et al. | Accommodation: Paying (55.4%), not paying (42.3%), other (2.3%), missing (1.5%) |
|  | Woodhead et al. | Owned/part owned (9.6%) Rented (71.5%), rent free (18.8%) |
|  | Yilmaz et al. | Council rented (6.6%), housing association rented (4.6%), private rented (4.3%), owner-occupied (78/5%), other (6.0%) |
| **Race, ethnicity, culture, language** | **Ethnicity** | |
|  | Woodhead et al. | White (45.1%), Black/Black British/mixed (38.2%), Other (6.8%) |
|  | Band et al. | White (92.3%), other (7.7%) |
|  | Bower et al. | White (97.6%), non-white (1.8%) |
|  | Howel et al. | White (99.2%) |
|  | Walters et al. | White British (88.2%), Other white (7.8%), African (2.0%), Other Asian (2.0%) |
|  | Yilmaz et al. | White (86.1%), Mixed (1.3%), Asian (5.6%), Black (3.6%) |
|  | Husain et al | Indian (24.5%), Bangladeshi (17.7%), Pakistani (55.2%), other South Asian (2.6%) |
|  | Forsyth et al. | White (90.0%), Black (3.2%), Asian (3.2%), other (3.8%) |
|  | Cheshire et al. | Black and Ethnic minorities (67.9%) |
|  | Stuttard et al. | White British (95.7%), all other (4.2%) |
|  | Coulton et al. | White (98%) |
|  | **Country of birth** | |
|  | Walters et al | UK (84.3%), another country (15.7%) |
|  | Language |  |
|  | Yilmaz et al. | English (86.1%), other (13.9%) |
|  | Husain et al. | English speaking: yes (93%), no (7%) |
| **Occupation** | **Employment status** | |
|  | Woodhead et al. | Unemployed (21.2%), employed (20.0%), retired (13.5%), outside labour force (45%) |
|  | Band et al. | Full-time (7.7%), part-time (9.0%), retired (48.0%), unemployed (9.6%), unable to work (22.3%), education or training (1.7%), carer (0.2%), missing (1.5%) |
|  | Bowel et al. | Retired or not economically active (93.4%), working or other (4.8%) |
|  | Howel et al. | Employed (11.4%), unemployed (4.6%), retired (73.4%), other (10.2%), missing (1.5%) |
|  | Walters et al. | Current employment: none (96.1%), part-time (3.9%) |
|  | Husain et al. | Full-time (12%), part-time (13.8%), unemployed (11.4%), sick (0.1%), home maker (53.4%), student (0.7%), other (8.5%) |
|  | Coulton et al. | Employed (11.0%) |
|  | **Occupation type** | |
|  | None reported |  |
| **Gender and sex** | **Sex** | |
|  | Woodhead et al. | Female (61.4%) |
|  | Band et al. | Female (70.4%), male (29.6%) |
|  | Bower et al. | Female 54.4% |
|  | Gathercole et al. | Female (58.5%), male (41.5%) |
|  | Howel et al. | Male (46.8%) |
|  | Walters et al. | Female (58.8%), male (41.2%) |
|  | Yilmaz et al. | Female (44%), male (66%) |
|  | Husain et al. | Female (100%) |
|  | Forsyth et al. | Male (100%) |
|  | Cheshire et al. | Female (66.6%) |
|  | Cockayne et al. | Female (65.5%), male (34.5%) |
|  | Stuttard et al. | Female (74.5%), male (25.5%) |
|  | Coulton et al. | Female (83.9%) |
|  | Ashburn et al. | Female (56.1), male (43.9%) |
|  | **Gender or gender identity** | |
|  | None reported |  |
|  | **Gender assigned at birth** | |
|  | None reported |  |
| **Religion** | **Religion** | |
|  | Husain et al. | Islam (88.1%), Hindu (7.1%), Christian (0.8%), Sikh (3.5%) Other (0.1%) |
| **Education** | **Educational attainment** | |
|  | Woodhead et al. | None (35.7%), Up to GCSE (28.7%), up to A level (21.6%), Degree + (14.3%) |
|  | Band et al. | Highest education level (primary 1.7%, secondary 4.1%, college 29.9%, missing 1.3%) |
|  | Bower et al. | College degree or higher (41.4%), school level qualifications (9.5%), no qualifications (43.9%) |
|  | Howel et al. | Primary (0.8%), secondary (84.4%), tertiary (14.8%) |
|  | Walters et al. | Educational level: <15 years (15.7%), aged 15-16 years (37.3), aged 17-20 years (9.8%), >21 years (37.3%) |
|  | Yilmaz et al. | Higher degree (11.1%), degree (18.9%), A level or equivalent (8.4%), HNC or HND or equivalent (7.1%), NVQ or equivalent (4.1%), GCSE (15.9%), School Leaving Certificate (18.6%), no formal qualifications (15.9%) |
|  | Husain et al. | Highest qualification: primary (3.7%) |
|  | Coulton et al. | Education after 16 (62.8%) |
|  | **Years of education** | |
|  | None reported |  |
|  | **Literacy** | |
|  | None reported |  |
|  | **SES category** | |
|  | None reported |  |
|  | **Individual or household income** | |
|  | Woodhead et al. | £0-549 (12.4%), £550-999 (6%), >£1000 (3.8%) |
|  | **Ability to manage on current income** | |
|  | Woodhead et al. | No housing payment problems (40.0%), housing payment problems (60.0%) |
|  | **Receipt of public assistance** | |
|  | Howel et al. | None (74.6%), financial (19.4%), non-financial (aids and adaptions) (4.2%), both financial and non-financial (1.5%), not known (0.3%), any type of benefit (22%) |
|  | **Deprivation index** | |
|  | Bower et al. | Mean index of multiple deprivation: 31.8 |
|  | Howel et al. | Mean IMD (28.7) range 3.2-74.8 |
|  | Walters et al. | Deprivation by postcode (IMD): 1-2 (most deprived) (4%), 3-4 (15.7%), 5-6 (23.5%), 7-8 (39.2%), 9-10 (17.6%) |
| **Social capital** | **Relationship status** | |
|  | Woodhead et al. | Single (42.3%), long-term relationship (25.5%), ex-relationship (32.5%) |
|  | Walters et al. | Single (13.7%), cohabiting (2%), married/cival partnership (37.3%), divorced (15.7%), widowed (31.4%) |
|  | Yilmaz et al. | Married or civil partnership (57.3%), divorced (5.6%), widowed (33.1%), single (1.7%), co-habiting (1.7%), or other (0.7%) |
|  | **Family size/structure** | |
|  | None reported |  |
|  | **Cohabitants** | |
|  | Woodhead et al. | Live alone (41.1%), lone parent (19.7%), live with others/family (16.5%) |
|  | Band et al. | Living alone: yes (51.6%), no (47.1%), missing (1.3%) |
|  | Bower et al. | Living alone: yes (51.6%), no (47.1%), missing (1.3%) |
|  | Gathercole et al. | Live in carer (48.5%), once daily carer (24.4%) less than once daily (27.0%) |
|  | Howel et al. | Living alone (47.0%) |
|  | Walters et al. | Living status: alone (51%), with spouse or partner (39.2%), with another family member (9.8%) |
|  | Yilmaz et al. | Living alone (27.8%), living with partner or spouse (52.6%), living with children (12.9%), other (6.6%) |
|  | Forsyth et al. | Single (13.7%), cohabiting (2%), married/civil partnership (37.5%), divorced (15.7%), widowed (31.4%) |
|  | Cockayne et al. | Living alone (48.5%), with friend of relative (5.2%), with partner or spouse (47.3%), in sheltered accommodation (2.6%) |
|  | Ashburn et al. | Lives alone (22.6%), with a spouse/partner (71.7%), with a friend/family (5.3%) |
|  | **Church/society memberships** | |
|  | None reported |  |
|  | **Significant others/social connectedness** | |
|  | Howel et al. | Emotional support: yes (94.4%), no (3.7%), missing (1.9%) |
| **Plus** | **Age** | |
|  | Woodhead et al. | 18-24 (1.6%), 25-34 (10.9%), 35-44 (18.5%), 45-45 (30.9%), 55-64 (26.0%), 65-74 (7.8%), 75+ (6.1%) |
|  | Band et al. | Mean (64.5) |
|  | Bower et al. | Mean (74.7) |
|  | Gathercole et al. | Mean (80.9) |
|  | Howel et al. | Mean (70.6) |
|  | Walters et al. | Mean (80.0) |
|  | Yilmaz et al. | Mean (79.9) |
|  | Husain et al. | Mean (31.3) |
|  | Forsyth et al. | Mean (58) |
|  | Cheshire et al. | Mean (64.3) |
|  | Cockayne et al. | Mean (80.1) |
|  | Stuttard et al. | Mean (48.7) |
|  | Coulton et al. | Mean (69.2) |
|  | Ashburn et al. | Mean (72) |
|  | **Chronic disease/disability** | |
|  | Woodhead et al. | Disabled/LTC (74.0%), not disabled (26.0%) |
|  | Bower et al. | All participants had one or more chronic health conditions |
|  | Gathercole et al. | 87.1% had a dementia diagnosis |
|  | Yilmaz et al. | Alzheimer's disease (46.0%), Vascular dementia (12.6%), lewy body dementia (3.3%), frontotemporal dementia (2.6%), other (27.8%), unable to specify (7.6%) |
|  | Cheshire et al. | 94% of participants identified themselves as having from one or more long-term health conditions (3 on average) |
|  | Cockayne et al. | Osteoperosis (15.3%), high blood pressure (45.6%), pain (50.4%), angina or heart troubles (21.6%), Parkinson's disease (1.7%), arthritis (RA/OA) (51.6%), anxiety or depression (12.8%), stroke (6.9%), urinary incontinence (19.2%), diabetes (17.6%), Ménière’s disease/conditions affecting balance/dizziness/vertigo (8.9%), chronic lung disease (6.6%), poor vision (19.6%), cancer (8.7%), other (37.6%) |
|  | Stuttard et al. | Long-term health condition? (expected to last more than 12 months): Yes (58.8%). All participants had severe/profound hearing loss |
|  | Ashburn et al. | Co-existing conditions: orthopaedic (50.2%), cardiovascular/respiratory (38.2%) |
|  | **Sexual orientation** | |
|  | None reported |  |
|  | **Immigration/residency status** | |
|  | None reported |  |
| **Features of relationships** | None reported |  |
| **Time-dependent relationships** | None reported |  |

Appendix H Supplementary Table 8: Summary table of critical appraisal judgements for included studies using JBI checklists (40)

| Study (Author, Year) | Q1 | Q2 | Q3 | Q4 | Q5 | Q6 | Q7 | Q8 | Q9 | Q10 | Q11 | Q12 | Q13 | Q14 | Q15 |
| --- | --- | --- | --- | --- | --- | --- | --- | --- | --- | --- | --- | --- | --- | --- | --- |
| Band et al. (2025) | Y | Y | Y | N | N | Y | Y | Y | Y | Y | Y | Y | N/A | N/A | N/A |
| Coulton et al. (2018) | Y | Y | Y | N | N | Y | Y | Y | Y | Y | Y | Y | N/A | N/A | N/A |
| Stuttard et al. (2021) | Y | Y | Y | N | N | Y | N | Y | Y | Y | Y | Y | N/A | N/A | N/A |
| Husain et al. (2025) | Y | Y | Y | N | N | Y | Y | Y | Y | Y | Y | Y | N/A | N/A | N/A |
| Bower et al. (2018) | Y | Y | Y | N | N | Y | Y | Y | Y | Y | Y | Y | N/A | N/A | N/A |
| Ashburn et al. (2019) | Y | Y | Y | N | N | Y | Y | Y | Y | Y | Y | Y | N/A | N/A | N/A |
| Cockayne et al. (2021) | Y | Y | Y | N | N | Y | Y | Y | Y | Y | Y | Y | N/A | N/A | N/A |
| Walters et al.(2017) | Y | Y | Y | N | N | Y | Y | Y | Y | Y | Y | Y | N/A | N/A | N/A |
| Yilmaz et al. (2024) | Y | Y | Y | N | N | Y | Y | Y | Y | Y | Y | Y | N/A | N/A | N/A |
| Gathercole et al. (2021) | Y | Y | N | N | N | Y | Y | Y | Y | Y | Y | Y | N/A | N/A | N/A |
| Forsyth et al. (2021) | Y | Y | Y | N | N | Y | Y | Y | Y | Y | Y | Y | N/A | N/A | N/A |
| Woodhead et al. (2017) | N/A | N/A | N/A | N/A | N/A | Y | N/A | Y | Y | Y | N/A | Y | Y | Y | Y |
| Cheshire et al. (2018) | Y | Y | Y | N | N | Y | Y | Y | Y | Y | Y | Y | N/A | N/A | N/A |
| Howel et al. (2019) | Y | Y | Y | N | N | Y | Y | Y | Y | Y | Y | Y | N/A | N/A | N/A |


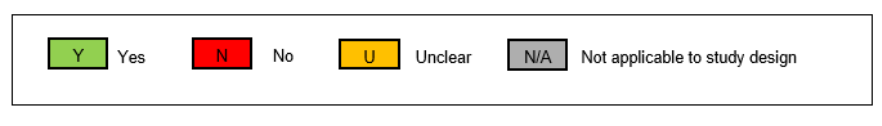


Appendix I Supplementary Table 9: Description of JBI critical appraisal checklist items and applicability of items by study design (40)

| Question # | Checklist item | Item included in study design checklist | |
| --- | --- | --- | --- |
|  |  | RCT | Quasi-experimental |
| 1 | Was true randomization used for assignment of participants to treatment groups? | ✓ | 🗶 |
| 2 | Was allocation to treatment groups concealed? | ✓ | 🗶 |
| 3 | Were treatment groups similar at the baseline? | ✓ | 🗶 |
| 4 | Were participants blind to treatment assignment? | ✓ | 🗶 |
| 5 | Were those delivering the treatment blind to treatment assignment? | ✓ | 🗶 |
| 6 | Were treatment groups treated identically other than the intervention of interest? | ✓ | ✓ |
| 7 | Were outcome assessors blind to treatment assignment? | ✓ | 🗶 |
| 8 | Were outcomes measured in the same way for treatment groups? | ✓ | ✓ |
| 9 | Were outcomes measured in a reliable way? | ✓ | ✓ |
| 10 | Was follow up complete and if not, were differences between groups in terms of their follow up adequately described and analysed? | ✓ | ✓ |
| 11 | Were participants analysed in the groups to which they were randomized? | ✓ | 🗶 |
| 12 | Was appropriate statistical analysis used? | ✓ | ✓ |
| 13 | Is it clear in the study what is the “cause” and what is the “effect” (i.e. there is no confusion about which variable comes first)? | 🗶 | ✓ |
| 14 | Was there a control group? | 🗶 | ✓ |
| 15 | Were there multiple measurements of the outcome, both pre and post the intervention/exposure? | 🗶 | ✓ |

Appendix J Table 11: Critical appraisal data summarising strengths and weaknesses of included studies

| **ID** | **Authors (Year)** | **Strengths** | **Weaknesses** |
| --- | --- | --- | --- |
| 1 | Band et al. (2025) | - Randomisation was stratified by organisation which kept groups balanced. - Characteristics between groups were similar at baseline. - Outcome assessors were not involved in the delivery of the intervention, reducing the risk of contamination between groups. | - Risk of detection and reporting bias as participants and facilitators were not blinded. - Outcome measures were self-reported, increasing the risk of recall or performance bias. - The sample lacked diversity (mostly female and White British), which limits generalisability. - High drop-out rates (20-30% for most outcomes at 6 months). |
| 2 | Coulton et al. (2018) | - Analysis was carried out blind to allocation, reducing detection bias. - Follow-up rates were 79% at 6 months, with similar drop-out rates in both groups. - Standardised, validated outcome measures were used which were relevant to the target population. - ITT analysis was performed which adjusted for baseline score, age, and gender. | - Participants self-referred, increasing the risk of selection bias. - Participants and facilitators were not blinded and outcomes were self-reported which may risk recall or performance bias. - The sample was not diverse (predominantly White British and female). |
| 3 | Stuttard et al. (2021) | - Data analysis was carried out blinded to group allocation, reducing the risk of detection bias. - ITT analysis was carried out with sensitivity analysis adjusting for baseline scores, age and gender. | - Participants and intervention assessors were not blinded, increasing risk of performance and detection bias. - The waitlist group also underwent an assessment so it was not a true no-intervention control. - All outcome measures were self-reported, increasing the risk of recall or performance bias. - Previous hearing dog applicants were excluded, therefore the findings may not be generalisable to all hearing loss populations. - The sample was not diverse (predominantly White British and female), potentially reducing generalisability. |
| 4 | Husain et al. (2025) | - Outcome assessors were blinded to treatment allocation. - Validated measurement tools were used, increasing reliability and comparability. - There were high retention rates (79% completed primary outcome measure at 6 months). - Appropriate statistical analysis was used (linear regression) adjusting for baseline scores and site of study. | - Participants and therapists were not blinded, increasing the risk of performance bias. - All outcomes were self-reported which increases the risk of reporting and recall bias. - Usual care may have varied between participants in the control group. - There was a relatively high attrition rate and missing data (23% missing data for primary outcome). - The intervention was targeted at a South Asian population therefore findings may not be generalisable to other populations. |
| 5 | Bower et al. (2018) | - Large sample size (n=1306). - There were similar baseline characteristics across both groups. - Outcome assessors were blinded to intervention allocation, reducing the risk of detection bias. - Both groups received standard care (the intervention group received CBT in addition to this). - High retention rates. | - Participants and facilitators were not blinded, increasing the risk of performance and detection bias. - A 6-month follow-up period may have been too short to detect meaningful changes in QOL or other longer-term outcomes. |
| 6 | Ashburn et al. (2019) | - Randomisation was computer-generated, stratified by site and baseline fall risk. - ITT analysis was carried out with multiple imputation for missing data. - Appropriate statistical analysis was used (logistic regression). | - Participants and therapists were not blinded, increasing the risk of performance bias. - The primary outcome (number of falls) was self-reported, increasing the risk of recall bias. - There was possible contamination from participants engaging in other physiotherapy or balance activities. - Differences in adherence to therapy and therapist effects were not controlled for. |
| 7 | Cockayne et al. (2021) | - Standardised and validated outcome measures were used, increasing the reliability and comparability of results. - Appropriate statistical analysis was used (logistic regression. - There was an adequate follow-up period to assess the long-term effects of intervention (12 months). | - Participants and therapists were not blinded, which may lead to performance bias. - All outcome measures were self-reported, increasing the risk of recall bias. - Only the home environment was risk-assessed as part of the intervention, however 25% of falls occurred outside of the home. |
| 8 | Walters et al.(2017) | - Outcome assessors were blinded to allocation, reducing the risk of detection bias. - There was a relatively large sample size (n=388) and the study took place across three regions, increasing generalisability. - There were high follow-up rates (86% at 12 months) with good uptake of the intervention. | - Participants were not blinded, increasing the risk of performance bias. - There was a possible ceiling effect for ADL as the Barthel Index scores were near maximum at baseline. - The duration of the intervention (6 months) may have been too short to demonstrate long-term functional effects. - Due to COVID-19 restrictions, some measures (gait speed, grip strength) were self-reported, increasing the risk of recall bias. - There was limited ethnic diversity in the sample, reducing generalisability. |
| 9 | Yilmaz et al. (2024) | - Randomisation was computer generated, with block stratification by selected variables, reducing the risk of confounding. - Baseline characteristics were similar between intervention and control groups. - There was good retention at 12 months. Dropout reasons were reported and evenly distributed across groups. | - Participants and facilitators were not blinded, increasing the risk of performance and detection bias. - Outcome measures at 24 months were limited to reduce assessment burden, however this means there was limited data available at this time point. - Participants were excluded if their carer could not access the intervention in English, increasing the risk of selection bias. - Primary outcome was self-reported, increasing the risk of recall bias. - The DAD (Disability Assessment for Dementia) scale is designed for mild-to-moderate dementia, but most participants had severe dementia, which may limit the validity of the results. |
| 10 | Gathercole et al. (2021) | - Recruitment took place in multiple settings reducing risk of selection bias. - Outcome assessors were blinded to group allocation, reducing the risk of detection bias. - Included a relatively large sample size and had a long follow-up period (up to 3 years). - ITT analysis was performed, adjusting for baseline differences. | - Participants and facilitators were not blinded, potentially introducing performance bias. - The intervention was tailored to individual need, making it difficult to standardise or identify which particular components were effective. - There was a baseline difference in primary outcome measurement scores between groups, although this was adjusted for in the analysis. - Relatively high attrition rates (28%), but this was similar across groups. - Potential confounding factors such as social support and healthcare access were not controlled for. |
| 11 | Forsyth et al. (2021) | - Analysis was performed blinded to allocation, reducing potential detection bias. - The study took place across 10 prisons, increasing generalisability. - Follow-up lasted approximately 2 years, providing long-term data. - ITT analysis was conducted, adjusting for baseline differences. | - Participants and facilitators were not blinded, which may have introduced performance bias and increased the risk of contamination in the contained environment of a prison. - High attrition rates due to the nature of the prison setting and participant transfers. - The initial assessment was delayed, with an average completion time of 20 days instead of the intended 1–2 weeks after arrival at prison. - The consistency of delivery of the intervention was affected by staffing shortages, high staff turnover, and operational disruptions. |
| 12 | Woodhead et al. (2017) | - The study was conducted over multiple sites, increasing external validity. - Appropriate statistical methods (logistic regression) were used, with potential confounders adjusted for in the analysis. - Subgroup analyses were conducted to explore potential differences in effects. | - The non-randomised design limits the ability to draw causal conclusions. - Short follow-up duration (3 months). - Self-reported outcome measures increase the risk of recall and reporting bias. |
| 13 | Cheshire et al. (2018) | - Computer-generated randomisation was used with stratification by general practice. - ITT analysis was conducted, adjusting for baseline differences. - Long follow-up period (18 months). | - Participants and intervention assessors were not blinded, increasing risk of performance and detection bias. - There was a high attrition rate (25% at 24 months), risking attrition bias and reducing statistical power. |
| 14 | Howel et al. (2019) | - Randomisation was computer-generated and stratified by general practice. - The study was conducted across 12 locations, improving generalisability. - Outcome assessors were blinded to group allocation, reducing assessment and detection bias. | - Participants and facilitators were not blinded, which may have introduced performance bias. - Most participants self-referred, increasing the risk of selection bias. - The intervention was aimed at a deprived population, but the sample was less deprived than expected, therefore the results may underestimate the effect of the intervention on more deprived populations. - There was a high attrition rate with differences between groups. - All outcome measures were self-reported, increasing the risk of recall bias. |
